# G9a deficiency activates TMEM27 to promote ferroptosis and enhances radiosensitivity in head and neck squamous cell carcinoma

**DOI:** 10.1038/s41420-025-02805-1

**Published:** 2025-11-10

**Authors:** Junli Hu, Yuanzheng Qiu, Wenhui Yuan, Guo Li, Donghai Huang, Xin Zhang, Yong Liu, Shanhong Lu, Chao Liu

**Affiliations:** 1 Department of Otolaryngology Head and Neck Surgery, Xiangya Hospital, Central South University, Changsha, China; 2 Department of Otolaryngology Head and Neck Surgery, Yantian District People’s Hospital, Shenzhen, Guangdong China; 3 Otolaryngology Major Disease Research Key Laboratory of Hunan Province, Changsha, China; 4 Clinical Research Center for Pharyngolaryngeal Diseases and Voice Disorders in Hunan Province, Changsha, China; 5 National Clinical Research Center for Geriatric Disorders, Xiangya Hospital, Central South University, Changsha, China

**Keywords:** Cancer epidemiology, Epidemiology

## Abstract

Epigenetic regulation, which modulates gene expression without altering the DNA sequence, plays a critical role in adaptive responses to environmental stressors, including irradiation. In this study, we investigated the role of histone H3K9 methylation in radiotherapy for head and neck squamous cell carcinoma (HNSCC). We identified G9a, a key lysine methyltransferase, as a critical regulator of H3K9me2 methylation in HNSCC radiotherapy. Inhibition of G9a using the small molecule inhibitor BRD4770 significantly enhanced radiosensitivity in HNSCC cells. Ferroptosis, a recently discovered form of iron-dependent cell death driven by lipid peroxidation, was found to play a pivotal role in radiotherapy-induced cell death and tumor suppression. RNA sequencing and KEGG pathway analysis revealed that G9a knockout increased radiosensitivity primarily by inducing ferroptosis. Further screening identified TMEM27 as a downstream target gene, and ChIP-qPCR confirmed that G9a binds to the H3K9me2 site of TMEM27, regulating its transcription. Importantly, we demonstrated that G9a-mediated expression of TMEM27 depends on its histone methyltransferase activity. In summary, this study reveals that G9a deficiency enhances radiosensitivity in HNSCC by activating TMEM27 to promote ferroptosis, providing a novel therapeutic strategy for overcoming radiotherapy resistance.

## Introduction

Head and neck squamous cell carcinoma (HNSCC) is the seventh most common cancer worldwide, with ~900,000 new cases and 450,000 deaths annually [1, 2]. Despite advances in treatment modalities such as radiotherapy, surgery, chemotherapy, targeted therapy, and immunotherapy, the clinical efficacy remains unsatisfactory, with a 3-year local recurrence rate of around 50% [3]. Radiotherapy, a cornerstone of HNSCC treatment, is recommended for early-stage HNSCC and is a critical component of salvage or combination therapy for advanced and recurrent cases [4]. However, the resistance of cancer cells to radiotherapy remains a significant barrier to achieving optimal treatment outcomes. Thus, an improved understanding of the underlying mechanisms of radiosensitivity and rational targeted strategies are urgently needed to improve therapeutic efficacy and patient outcomes in HNSCC. Targeted radiotherapy aims to enhance the precision of radiation delivery to tumor tissues while minimizing damage to normal tissues. This approach includes combining radiotherapy with targeted therapies such as monoclonal antibodies, small molecule inhibitors, gene therapy, and chemotherapy drugs to increase radiosensitivity [5]. Understanding the molecular mechanisms underlying radiosensitivity is crucial for developing effective strategies to overcome resistance and improve patient outcomes.

Cell death mechanisms, including apoptosis, necrosis, pyroptosis, autophagy, and ferroptosis, play critical roles in cancer therapy. Ferroptosis, a recently discovered form of regulated cell death, is characterized by iron-dependent lipid peroxidation and distinct morphological changes in mitochondria [6]. Unlike apoptosis, ferroptosis has been shown to play a vital role in the antitumor effects of radiotherapy, particularly in apoptosis-resistant cells [7, 8]. Hence, targeting ferroptosis may bypass the limitations of apoptosis resistance and enhance the sensitivity of tumor cells to radiotherapy.

Epigenetics, which regulates gene expression without altering the DNA sequence, plays a critical role in cancer development and progression. Epigenetic modifications, including DNA methylation, histone covalent modifications, chromatin remodeling, and noncoding RNA changes, influence gene expression and activity [9, 10]. The earliest research on the relationship between epigenetics and cancer was based on gene expression and DNA methylation [11]. In recent years, histone modification [12], which is closely related to DNA repair, has attracted increasing attention. Histone H3 lysine 9 (H3K9) methylation, a key epigenetic marker associated with heterochromatin formation and transcriptional silencing, has been implicated in the growth and progression of various cancers [13–15]. G9a, a histone methyltransferase responsible for H3K9 methylation, is a potential therapeutic target due to its role in cancer cell survival and resistance.

TMEM27 (Transmembrane Protein 27, CLTRN) is a less well-studied protein, but it is known to play roles in cellular processes such as metabolism, signaling, and potentially cell death regulation. TMEM27 has been implicated in glucose metabolism and insulin secretion in pancreatic β-cells, suggesting a role in metabolic homeostasis [16–18]. As a transmembrane protein, TMEM27 may participate in signal transduction pathways, though its specific signaling mechanisms remain unclear. While not directly linked to ferroptosis, TMEM27’s involvement in cellular stress responses and signaling pathways suggests it could play a role in regulating cell death mechanisms.

This study aims to investigate whether G9a deficiency activates TMEM27 to promote ferroptosis and enhance radiosensitivity in HNSCC. By exploring the interplay between G9a, TMEM27, and ferroptosis, we seek to identify novel therapeutic strategies to improve radiotherapy outcomes in HNSCC patients.

## Results

### Radiation-induced H3K9 methylation is regulated by the histone methyltransferase G9a

**Fig. 1:**
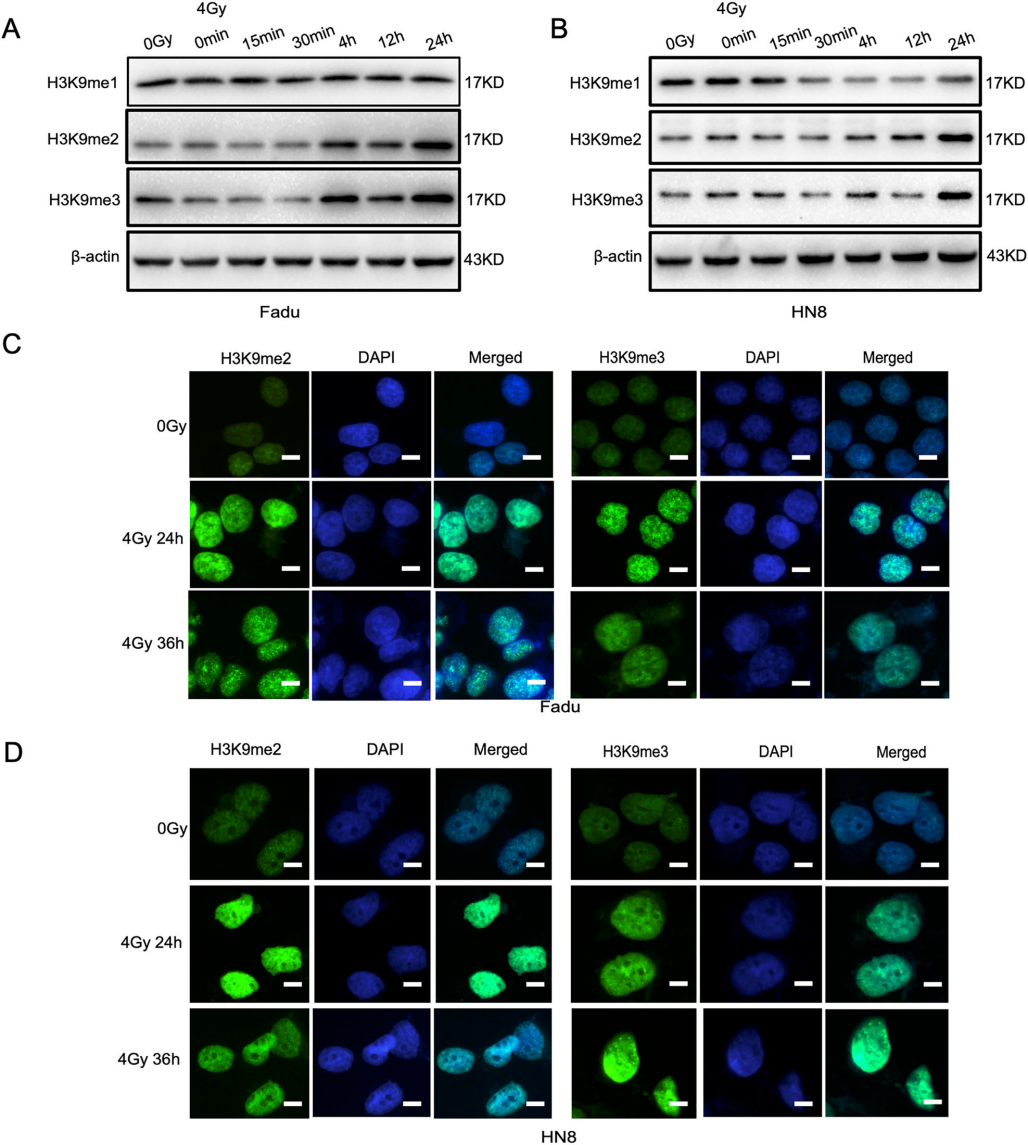
Changes in histone H3K9 methylation sites in HNSCC cells after 4 Gy irradiation and 24 h incubation. **A**, **B** Following 4 Gy irradiation of Fadu or HN8 cells, protein levels at the H3K9me1 sites remained unchanged, while levels at the H3K9me2 and H3K9me3 sites showed significant alterations. **C**, **D** Immunofluorescence analysis of HNSCC cells after 4 Gy irradiation and incubation for 24 h and 36 h revealed increased expression levels of H3K9me2 and H3K9me3 in the nucleus.

To investigate the effects of irradiation on histone H3K9 methylation, HNSCC cells (Fadu and HN8) were irradiated with a dose of 4 Gy, consistent with our previous studies [19–21]. Changes in H3K9 methylation were assessed by Western blot analysis at various time points (0 min, 15 min, 30 min, 4 h, 12 h, and 24 h) post-irradiation. In both Fadu and HN8 cells, significant increases in H3K9me2 and H3K9me3 protein levels were observed at 24 h, while H3K9me1 levels remained unchanged (Figs. 1A, B and S1C). Similar trends in H3K9me2 expression were observed over a 72-h period, with the most pronounced increase occurring at 24 h (Fig. S1A, S1B, and S1D). These findings were further validated by immunofluorescence staining in Fadu and HN8 cells (Figs. 1C, D and S1E).

**Fig. 2:**
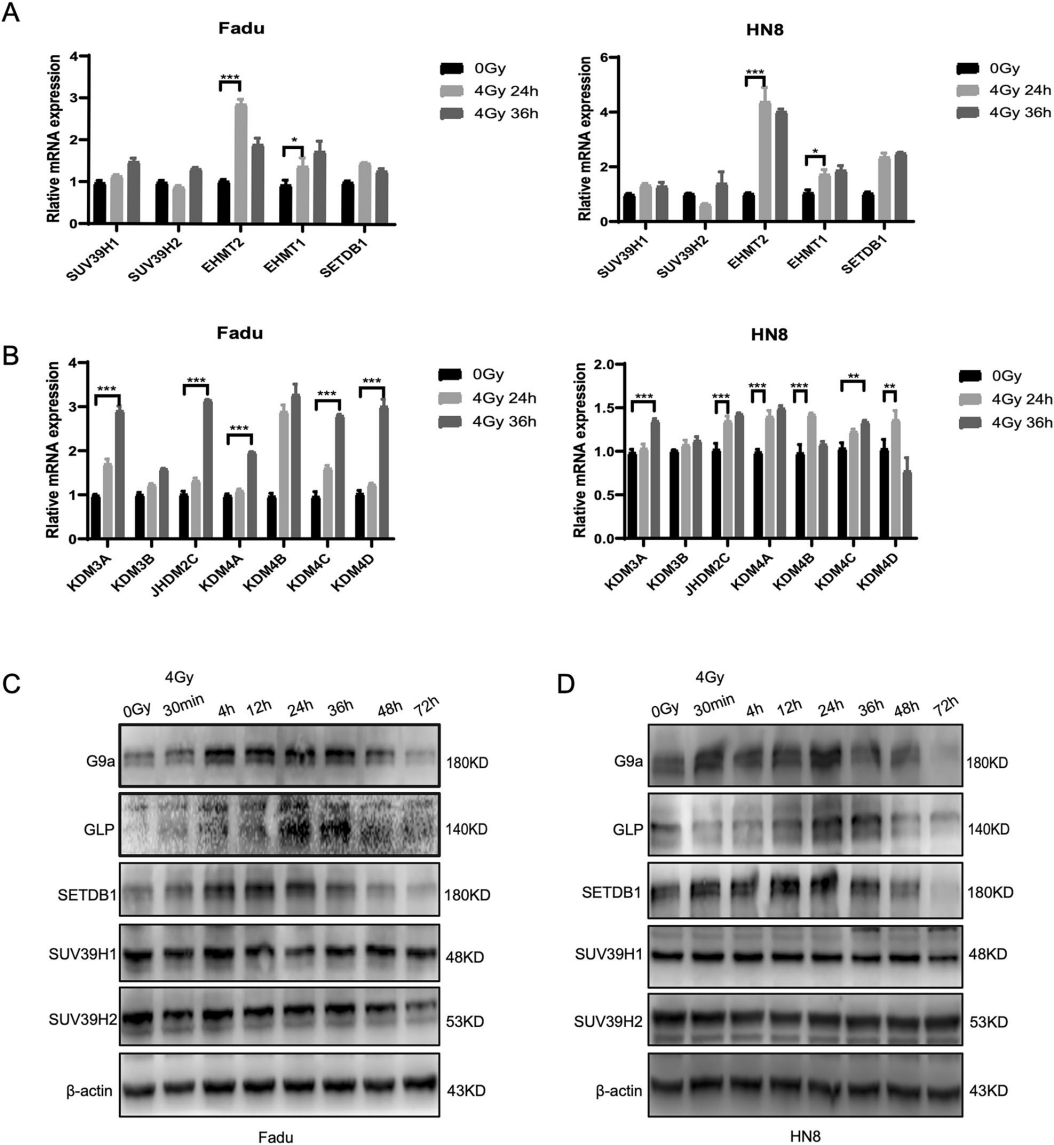
Screening for methyltransferases and demethyltransferases regulating H3K9 methylation in HNSCC Cells after 4 Gy irradiation. **A** qPCR analysis was performed to assess changes in H3K9 methyltransferase mRNA levels in Fadu and HN8 cells after 4 Gy irradiation and incubation for 24 h and 36 h. **B** qPCR analysis was also used to evaluate changes in H3K9 demethyltransferase mRNA levels under the same conditions. Statistical significance is indicated as *p < 0.05, **p < 0.01, and ***p < 0.001. Western blot analysis was conducted to verify the expression levels of H3K9 methyltransferase proteins within 72 h after 4 Gy irradiation in Fadu (**C**) and HN8 (**D**) cells.

Histone H3K9 methylation is tightly regulated by lysine methyltransferases (KMTs) and demethylases (KDMs) to maintain genomic stability. We summarized the regulatory roles of KMTs and KDMs in H3K9 methylation (Fig. S2A). To explore the mechanism underlying irradiation-induced H3K9 methylation, we analyzed the mRNA expression levels of KMTs and KDMs in Fadu and HN8 cells following 4 Gy irradiation using qPCR. The results revealed significant upregulation of the methyltransferases EHMT2 (G9a) and EHMT1, with EHMT2 showing the most pronounced increase (Fig. 2A, B). Consistent with these findings, Western blot analysis of protein extracts collected at various time points (0 min, 30 min, 4 h, 12 h, 24 h, 36 h, 48 h, and 72 h) demonstrated that the protein expression levels of G9a and GLP correlated with their mRNA levels and the observed changes in H3K9 methylation (Figs. 2C, D and S2B). Based on these results, we concluded that irradiation-induced increases in H3K9 methylation are primarily regulated by methyltransferases, with EHMT2 (G9a) playing a pivotal role.

### Inhibition of G9a with BRD4770 enhances the efficacy of radiotherapy in HNSCC in vitro and in vivo

**Fig. 3:**
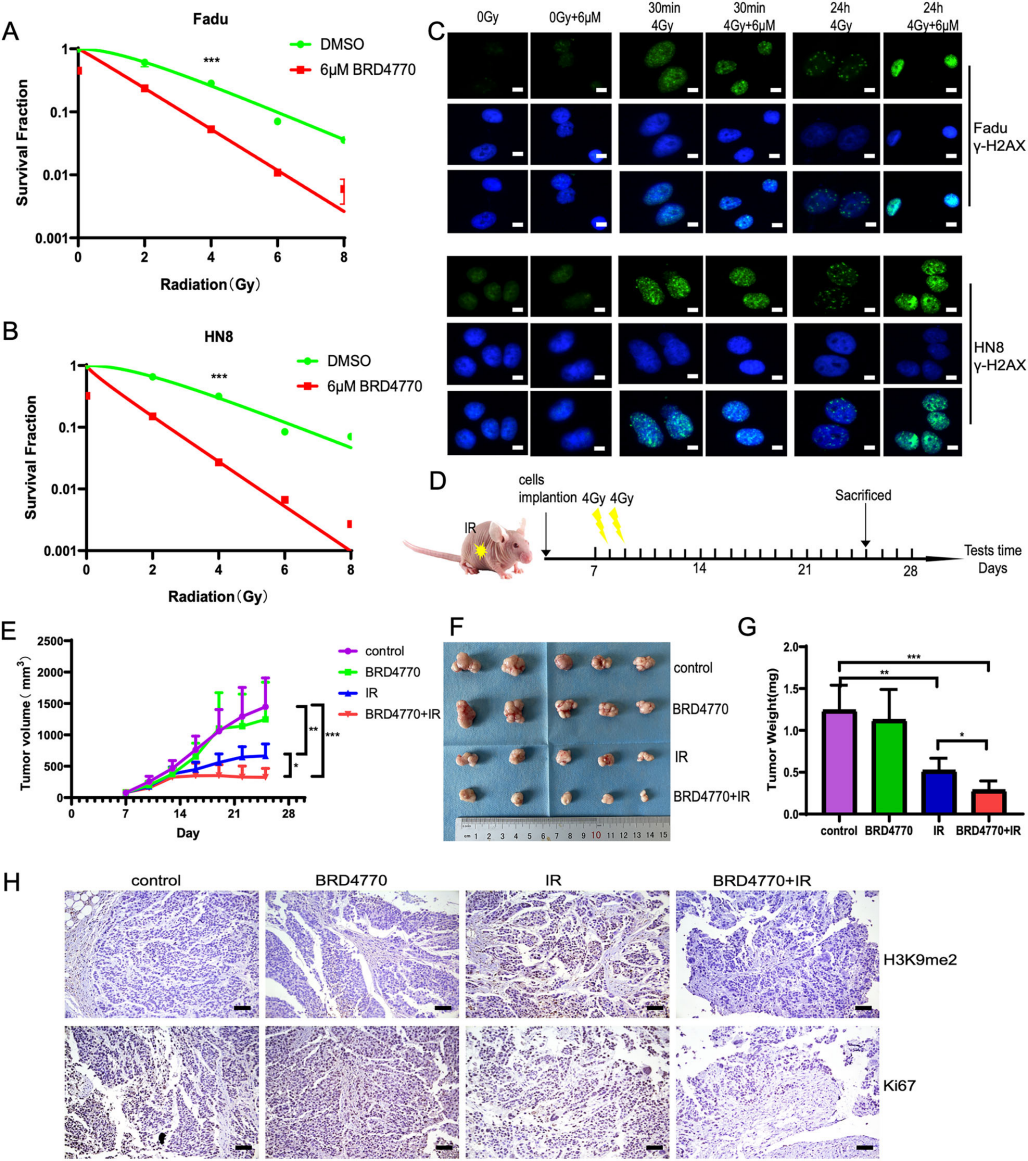
BRD4770 regulates histone H3K9 methylation and enhances the radiosensitivity of HNSCC cells in vitro and in vivo. **A**, **B** Fadu and HN8 cells were treated with 6 μM BRD4770 for 4 h and then irradiated with 0–8 Gy. Survival curves were generated using a multiple-target single-hit model. Two-way ANOVA was used to compare the survival curves between DMSO and BRD4770 groups (p < 0.001). **C** Fadu and HN8 cells were treated with 6 μM BRD4770 for 4 h, irradiated with 4 Gy, and fixed at 30 min and 24 h post-irradiation. Nuclei were stained with DAPI, and γ-H2AX foci (green) were visualized to assess DNA damage; scale bar, 10 μm. **D** Schematic of the in vivo experimental design for drug administration and radiotherapy in nude mice. The timeline begins at tumor seeding (Day 0). The yellow star indicates the tumor site and radiotherapy target, and the yellow lines represent radiotherapy sessions. **E**, **F** Tumor growth differences were analyzed using a mixed-effects linear model, and photographs of tumors from each group were taken (n = 5). Statistical significance is indicated as * p < 0.05, ** p < 0.01, and *** p < 0.001. **G** Effects of BRD4770 combined with IR on tumor weight in nude mice. **H** IHC analysis of H3K9me2 and Ki67 expression levels in tumor tissues from treated mice; scale bar, 100 μm.

To explore the role of G9a on radiotherapy of HNSCC, different concentrations of the specific G9a inhibitor BRD4770 were applied to treat Fadu and HN8 cells, and the IC50 value was calculated to be ~6 µM by CCK8 (Fig. S2C). Then the Fadu and HN8 cells were treated with BRD4770 following radiation, which showed that inhibition of G9a with BRD4770 enhanced the efficacy of radiotherapy in HNSCC (Fig. 3A, B). Ionizing radiation induces double-strand DNA breaks (DSBs), leading to cell damage and death. The phosphorylation of histone H2AX to γ-H2AX serves as a key marker of DSBs. In this study, Western blot analysis revealed that γ-H2AX expression peaked at 30 min post-irradiation but decreased by 24 h (Fig. S3A), reflecting the dynamic repair of DSBs. Notably, combined treatment with BRD4770 and irradiation significantly increased γ-H2AX expression compared to irradiation alone, as confirmed by both immunofluorescence and Western blot analysis (Fig. 3C, S3A, and S3C). Additionally, BRD4770 treatment enhanced the expression of DSB repair marker 53BP1 foci following irradiation (Fig. S3B and S3D). Since G9a primarily catalyzes the di-methylation of H3K9 [22], immunofluorescence further demonstrated that BRD4770 treatment also attenuated irradiation-induced H3K9me2 methylation (Fig. S3E). These findings suggest that G9a inhibition promotes the DSBs and radiosensitization in HNSCC in vitro.

To validate these results in vivo, Fadu cells were implanted into the right upper arms of nude mice (Fig. 3D). When tumor volumes reached 80–100 mm³, the mice were randomized into four groups: control, BRD4770 alone, irradiation (IR) alone, and BRD4770 combined with IR. BRD4770 was administered intraperitoneally every other day, and mice received two doses of 4 Gy irradiation following the first injection. Tumors were monitored for 25 days post-implantation. While BRD4770 alone did not significantly affect tumor growth, it markedly sensitized Fadu tumors to a total of 8 Gy irradiation, as evidenced by reduced tumor size and weight (Fig. 3E–G). Immunohistochemical analysis confirmed that BRD4770 reduced IR-induced H3K9me2 methylation levels in tumor tissues, consistent with the in vitro results (Figs. 3H and S4G). Furthermore, the combination of BRD4770 and IR significantly decreased Ki67 expression, indicating inhibition of tumor proliferation (Figs. 3H and S4G). These results demonstrate that G9a inhibition and subsequent reduction of H3K9me2 methylation sensitize HNSCC to radiotherapy.

### G9a deficiency in HNSCC enhances the sensitivity of HNSCC to radiotherapy

**Fig. 4:**
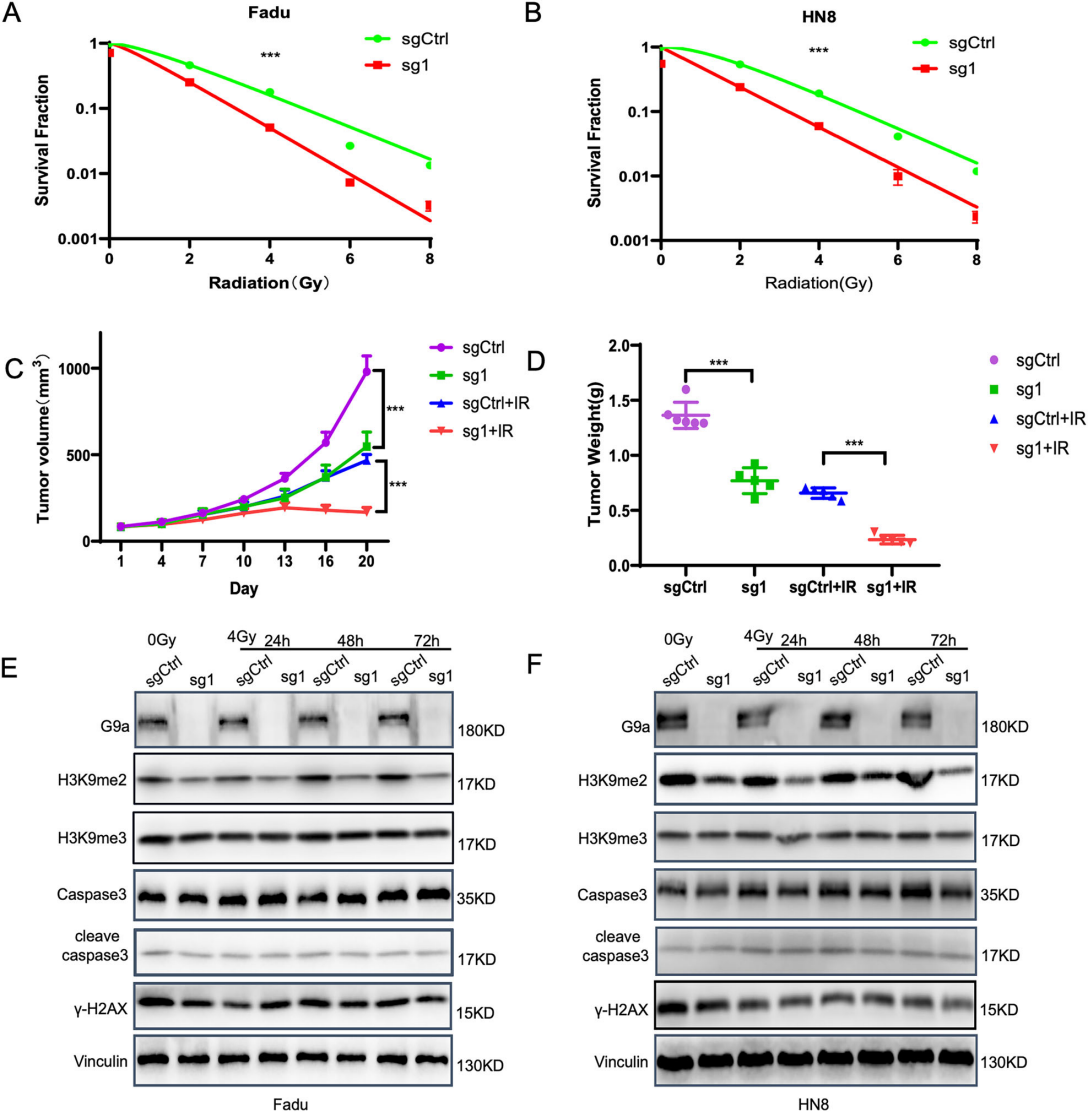
Effect of G9a knockout on the radioresistance of HNSCC cells. **A**, **B** Fadu and HN8 cells from the sgCtrl (control) and sg1 (G9a knockout) groups were irradiated with 0–8 Gy. Survival curves were generated using a multiple-target single-hit model, and two-way ANOVA was used to compare the survival curves between the sg1 and sgCtrl groups (p < 0.001). **C**, **D** Tumor growth differences were analyzed using a mixed-effects linear model. The effects of G9a knockout (KO) combined with IR on tumor weight were evaluated (n = 5, p < 0.001). **E**, **F** The G9a Control (sgCtrl) and G9a KO (sg1) groups were treated with radiotherapy, followed by irradiation (0 Gy and 4 Gy). Proteins were extracted at designated time points (24 h, 48 h, and 72 h), and Western blot was used to assess the expression levels of G9a, H3K9me2, H3K9me3, γ-H2AX, and apoptosis-related proteins.

To further investigate the radiosensitizing effects of G9a, we generated G9a knockout (KO) Fadu and HN8 cells using CRISPR-Cas9 technology with two independent single guide RNAs (sgRNAs) (Fig. S4A). Monoclonal G9a KO cells were selected, expanded, and validated by Western blot (Fig. S4C, S4D). Sanger sequencing confirmed homozygous knockout of G9a using sg1 (Fig. S4B). Western blot analysis demonstrated that G9a knockout significantly reduced H3K9me2 levels in both Fadu and HN8 cells (Fig. S4C, S4D, S4E, and S4F). To assess the impact of G9a deficiency on radiosensitivity, clonogenic survival assays were performed on G9a KO and control cells following irradiation (0–8 Gy). G9a knockout markedly inhibited the clonogenic ability of both Fadu and HN8 cells compared to controls (Fig. 4A, B), indicating enhanced radiosensitivity.

To validate these findings in vivo, G9a KO and control Fadu cells were used to induce subcutaneous tumors in nude mice. When tumor volumes reached 80 mm³, mice were irradiated with 4 Gy on two consecutive days and sacrificed 20 days later. All tumor-bearing mice tolerated radiotherapy without significant changes in body weight. Consistent with the in vitro results, G9a knockout significantly inhibited tumor growth and enhanced tumor sensitivity to radiation (Fig. 4C, D).

### G9a knockout induced ferroptosis in HNSCC cells

To elucidate the mechanism underlying G9a knockout-induced radiosensitization in HNSCC cells, we collected protein samples from G9a control (sgCtrl) and G9a knockout (sg1) groups at 0 and 4 Gy irradiation, followed by analysis at 24, 48, and 72 h. Western blot confirmed the successful silencing of G9a and downregulation of H3K9me2 in the G9a KO group post-irradiation. Notably, levels of H3K9me3, γ-H2AX, caspase-3, and cleaved caspase-3 in the G9a KO group showed no significant changes compared to the control group at any time point (Figs. 4E, F, S5A and S5B), suggesting that G9a knockout induces cell death independently of caspase-3-mediated apoptosis.

**Fig. 5:**
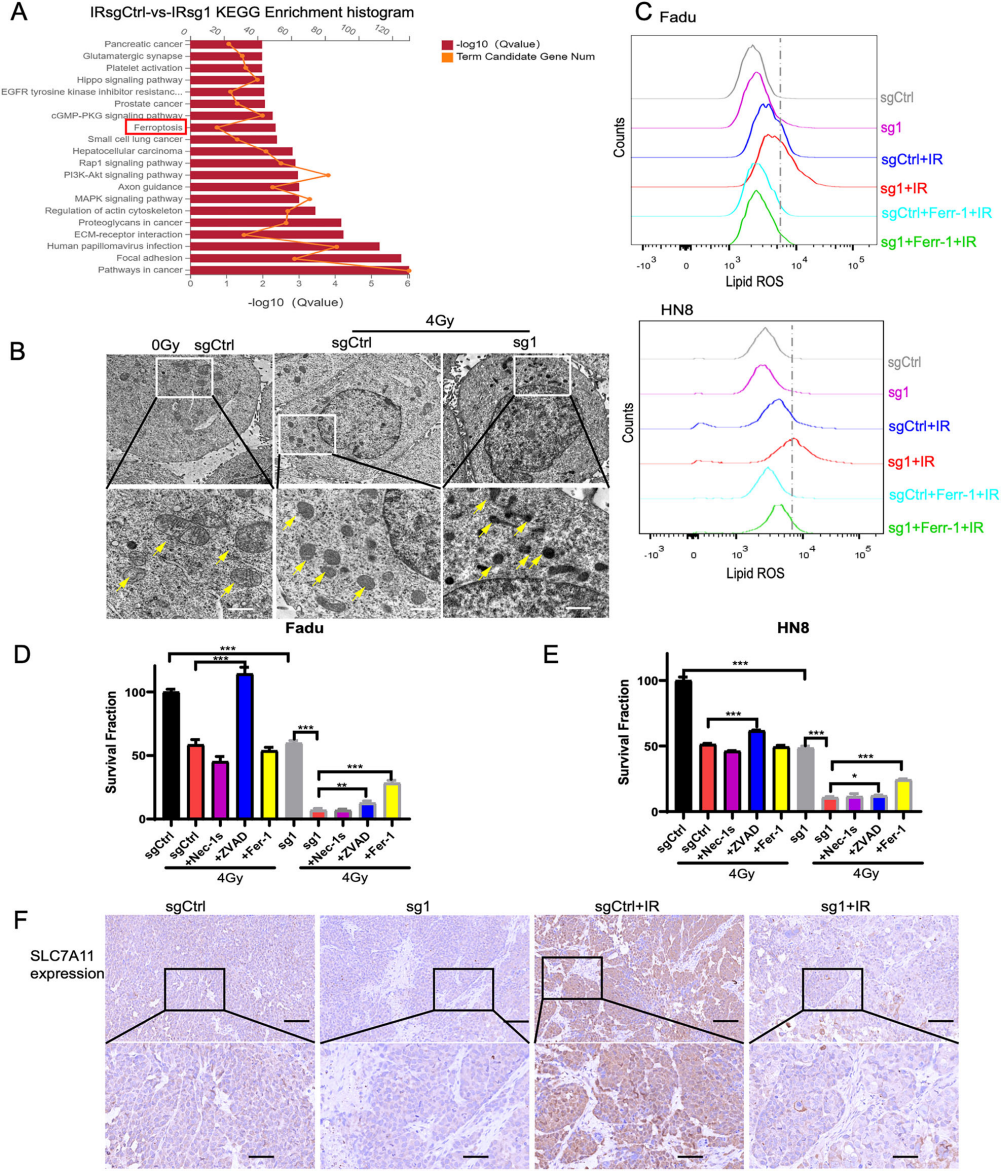
Radiotherapy-induced ferroptosis in G9a-knockout HNSCC cells. **A** RNA-seq results showing the top 20 enriched KEGG pathways in the Fadu sgCtrl and sg1 (G9a knockout) groups after 4 Gy irradiation for 24 h. **B** Transmission electron microscopy (TEM) images revealing changes in mitochondrial morphology before and after radiotherapy in the Fadu sgCtrl and sg1 groups. **C** Changes in reactive oxygen species (ROS) levels were detected using the ferroptosis inhibitor Fer-1 and the C11-BODIPY probe in the sgCtrl and sg1 groups of Fadu and HN8 cells after irradiation with 0 Gy and 4 Gy. **D**, **E** Fadu and HN8 cells in the sgCtrl and sg1 groups were treated with Nec-1s (necroptosis inhibitor), ZVAD (apoptosis inhibitor), and Fer-1 (ferroptosis inhibitor) for 24 h, followed by irradiation (0 Gy or 4 Gy). Cell survival rates were determined by colony formation assays after 14 days of culture. **F** IHC analysis of the effect of sg1 combined with radiotherapy on the expression of SLC7A11 in tumor tissues from mice; scale bars, 100 μm. Statistical significance is indicated as *p < 0.05, **p < 0.01, and ***p < 0.001.

To further investigate the transcriptomic changes associated with G9a knockout, RNA sequencing was performed on mRNA extracted from G9a control (sgCtrl) and G9a KO (sg1) Fadu cells after 4 Gy irradiation. KEGG pathway enrichment analysis identified ferroptosis as one of the key pathways linked to G9a-induced cell death (Fig. 5A). Transmission electron microscopy (TEM) revealed that irradiated G9a-depleted Fadu cells exhibited shrunken mitochondria with increased membrane density, a hallmark morphological feature of ferroptosis (Fig. 5B). Additionally, radiation-induced lipid peroxidation and elevated reactive oxygen species (ROS) levels in G9a KO cells were significantly attenuated by the ferroptosis inhibitor ferrostatin-1 (Fer-1) (Figs. 5C and S5C).

To delineate the mode of cell death induced by G9a knockout, we treated irradiated G9a KO Fadu and HN8 cells with inhibitors targeting ferroptosis (Fer-1), apoptosis (Z-VAD-fmk, ZVAD), and necroptosis (necrostatin-1s, Nec-1s). Clonogenic survival assays demonstrated that Fer-1 and ZVAD partially rescued irradiation-induced cell death, with Fer-1 showing the most pronounced effect (Fig. 5D, E). Consistent with these in vitro findings, immunohistochemical (IHC) staining revealed decreased expression of SLC7A11, a key negative regulator of ferroptosis, in G9a KO tumors compared to controls following radiotherapy (Figs. 5F and S5D). Collectively, these results strongly suggest that ferroptosis is the primary mode of irradiation-induced cell death in G9a-deficient HNSCC cells.

### Depletion of histone methyltransferase G9a promotes HNSCC radiation sensitivity by epigenetic activation of the ferroptosis gene TMEM27

**Fig. 6:**
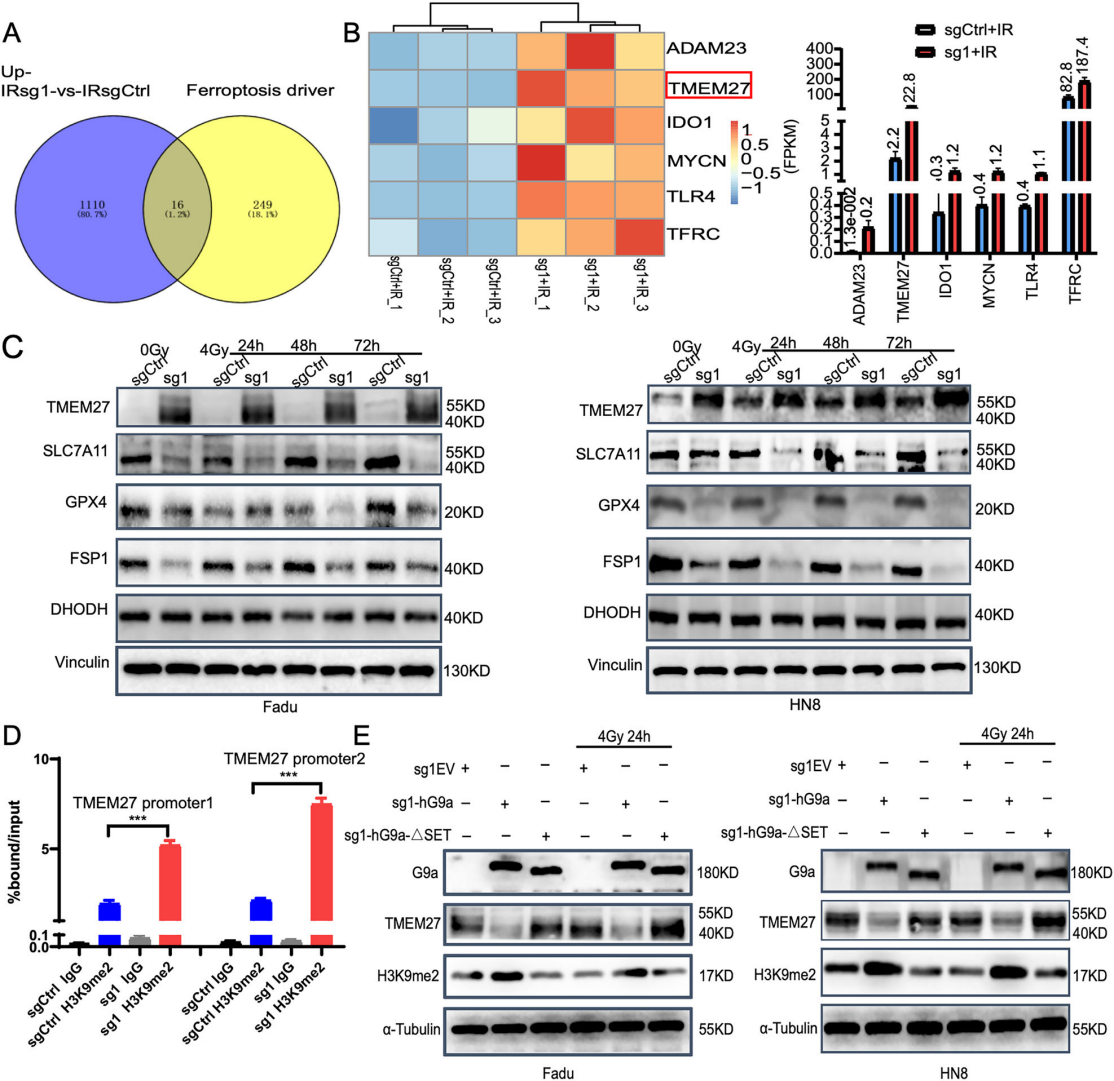
G9a knockout activates TMEM27 to promote ferroptosis, and G9a-mediated transcriptional repression of TMEM27 is HMTase dependent. **A**, **B** Differentially expressed genes after 4 Gy irradiation in the Fadu sgCtrl and sg1 (G9a knockout) groups were intersected with ferroptosis-related genes, leading to the identification of TMEM27 as a key candidate gene. **C** Proteins were collected at 24 h, 48 h, and 72 h after irradiation (0 Gy and 4 Gy) in the sgCtrl and sg1 groups of Fadu and HN8 cells. Western blot analysis revealed changes in TMEM27 protein levels and key proteins in the ferroptosis defense system. **D** ChIP-qPCR analysis showed that the G9a knockout (sg1) group exhibited reduced H3K9me2 levels in the TMEM27 promoter region after radiotherapy, leading to TMEM27 transcriptional activation and increased expression (***p < 0.001). **E** Western blot analysis was performed to assess changes in TMEM27 protein expression and ferroptosis defense system proteins in Fadu and HN8 cells transfected with sg1EV (empty vector), sg1-hG9a (wild-type G9a), and sg1-hG9a-ΔSET (G9a lacking the SET domain).

To identify candidate genes involved in ferroptosis regulation by G9a methylation following radiotherapy, we conducted RNA sequencing (RNA-Seq) screening. In the G9a knockout group after irradiation, upregulated genes were compared with ferroptosis activation-related genes from the Ferroptosis Database (http://www.zhounan.org/ferrdb/current/). This analysis identified 16 ferroptosis activation-related genes that were significantly upregulated (Fig. 6A), with TMEM27 showing the most pronounced increase in expression (Fig. 6B). Western blot analysis confirmed that TMEM27 expression was elevated in G9a knockout cells at baseline and further increased after irradiation. Conversely, the expression of ferroptosis protective factors, including SLC7A11, GPX4, and FSP1, was significantly reduced (Fig. 6C and S5E).

To explore the transcriptional regulation of TMEM27 by G9a, we performed chromatin immunoprecipitation (ChIP) analysis. A series of primers targeting the TMEM27 promoter region was designed to assess G9a binding and H3K9me2 methylation levels in Fadu and HN8 cells. ChIP-qPCR results demonstrated that H3K9me2 methylation in the TMEM27 promoter region was increased in G9a knockout cells after irradiation, leading to the activation of TMEM27 transcription (Fig. 6D).

To determine the role of G9a’s SET domain in regulating TMEM27 expression, we transfected G9a knockout cells with plasmids expressing either wild-type G9a (hG9a), which has methyltransferase activity, or a G9a mutant lacking the SET domain (hG9a-ΔSET), which lacks methyltransferase activity. Transfection with hG9a restored G9a and H3K9me2 expression and downregulated TMEM27 expression. In contrast, transfection with hG9a-ΔSET restored G9a expression but had no significant effect on H3K9me2 or TMEM27 levels (Fig. 6E and S5F). These findings demonstrate that G9a-mediated downregulation of TMEM27 depends on its histone methyltransferase (HMTase) activity.

To further clarify the role of TMEM27 on ferroptosis and radiosensitivity in HNSCC, we firstly detected the expression of TMEM27 at different time points post 4 Gy irradiation in HNSCC cells, which showed that the expression of TMEM27 was decreased at 12 h and 24 h following 4 Gy irradiation in HN8 cells (Fig. S6A). Then, following overexpression of TMEM27 in HNSCC cells (Fig. S6B), TEM analysis revealed that irradiated TMEM27-overexpressing Fadu cells exhibited shrunken mitochondria with increased membrane density (Fig. S6C) and colony formation assays demonstrated that overexpression of TMEM27 enhanced the efficacy of radiotherapy in Fadu and HN8 cells (Fig. S6D). Furthermore, radiation-induced lipid peroxidation and elevated reactive oxygen species (ROS) levels in TMEM27-overexpressed were significantly attenuated by the ferroptosis inhibitor ferrostatin-1 (Fig. S6E and S6F).

All these above data demonstrated that the depletion of G9a enhances radiation sensitivity in HNSCC by epigenetically activating TMEM27 and promoting ferroptosis.

### Clinicopathologic features of G9a in HNSCC

**Fig. 7:**
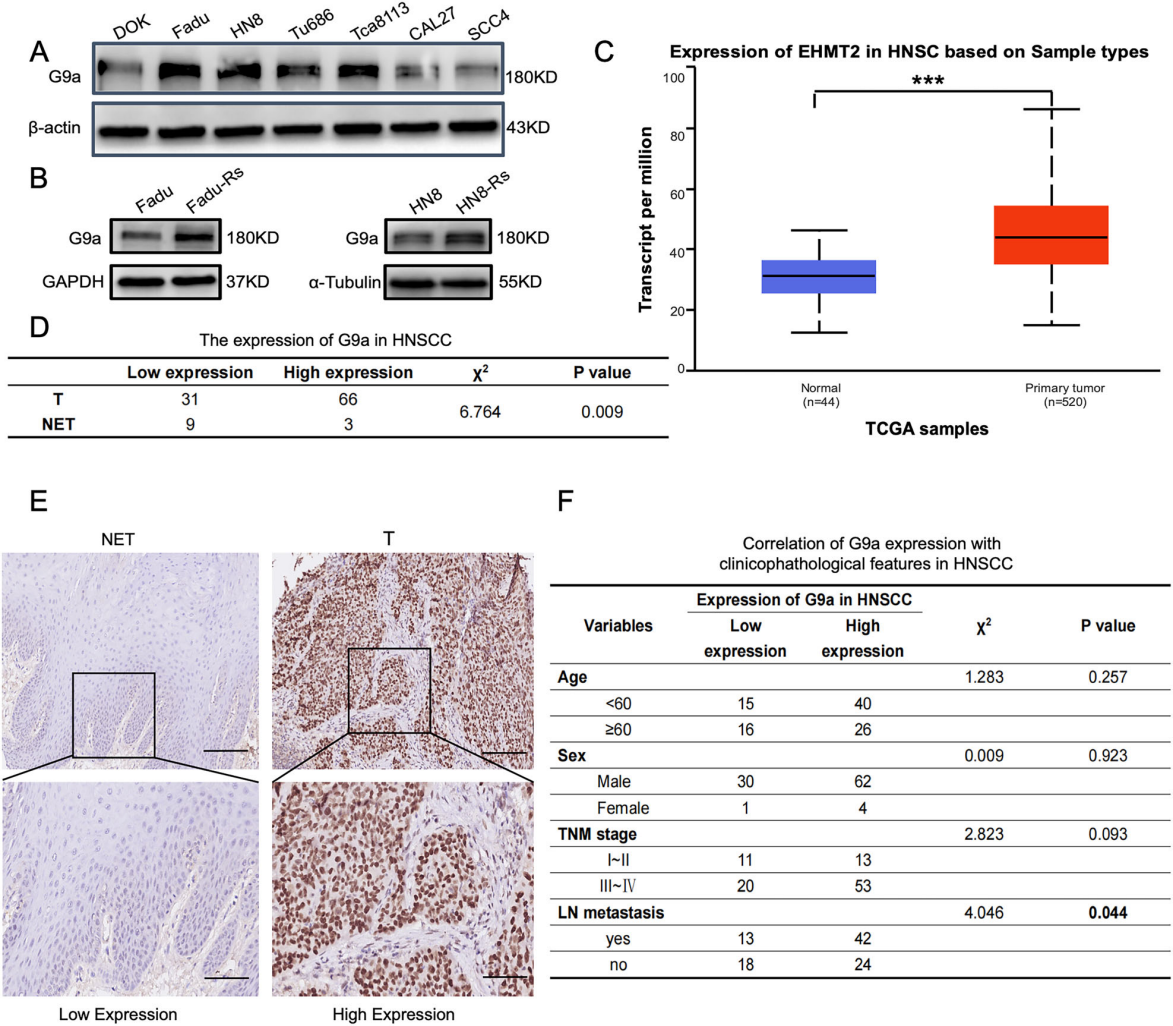
Clinicopathologic features of G9a in HNSCC. **A** Western blot analysis of G9a protein expression levels in human oral mucosal precancerous lesion cells and HNSCC cell lines. **B** Western blot analysis of G9a protein expression levels in parent and radioresistant HNSCC cells. **C** Analysis of G9a (EHMT2) mRNA expression in 520 HNSCC samples and 44 normal samples from the TCGA database, showing elevated G9a expression in HNSCC tissues. **D** IHC results demonstrating that G9a expression is significantly upregulated in HNSCC tissue samples compared to noncarcinoma epithelial tissues (NETs). **E** Representative IHC staining images showing high expression of G9a in HNSCC tissues and low expression in NETs. Scale bars: 100 μm (top), 50 μm (bottom). **F** Correlation of G9a expression with clinicopathological features in HNSCC. Statistical significance is indicated as * p < 0.05 and ***p < 0.001.

To assess the clinical significance of G9a in HNSCC, we first examined G9a protein levels in six human HNSCC cell lines. As expected, G9a protein levels were significantly higher in HNSCC cell lines compared to a human precancerous oral mucosa cell line (DOK) (Fig. 7A). Meanwhile, we detected the expression of G9a in the radioresistant Fadu and HN8 cells (Fadu-Rs, HN8-Rs), which showed that compared to the parent cells, G9a was upregulated in radioresistant cells (Fig. 7B). To further validate these findings, we analyzed G9a mRNA expression in the TCGA database, which includes 520 HNSCC tumor samples and 44 normal tissues. The RNA-seq data revealed that G9a mRNA levels were significantly elevated in HNSCC tissues compared to adjacent normal tissues (Fig. 7C). Next,we performed immunohistochemistry (IHC) to quantify G9a protein expression in 97 paraffin-embedded HNSCC tumor samples and 12 noncarcinoma epithelial tissues (NETs). G9a was predominantly localized in the nucleus of cancer cells, and its protein levels were markedly higher in HNSCC tissues compared to NETs (Fig. 7D, E). Clinically, high G9a expression was strongly associated with lymph node metastasis (Fig. 7F). However, no significant correlation was observed between G9a expression levels and clinical stage (Fig. 7F), which may be attributed to the limited sample size.

## Discussion

Radiation resistance in HNSCC remains a significant clinical challenge, and emerging evidence suggests that abnormal epigenetic modifications play a critical role in its development [23]. Epigenetic changes can serve as adaptive responses to protect cells from radiation-induced damage or drive toxic pathways, leading to adverse effects. In this study, we observed changes in histone H3K9 methylation following 4 Gy irradiation in parental HNSCC cells. While H3K9me1 levels remained unchanged, H3K9me2 and H3K9me3 levels increased significantly 24 h post-irradiation, coinciding with the timeframe of DNA damage repair. These findings suggest that H3K9me2 and H3K9me3 modifications may contribute to the development of radioresistance. Histone methylation is regulated by lysine methyltransferases (KMTs) and demethyltransferases (KDMs) [22], many of which are overexpressed in tumors and have emerged as promising therapeutic targets [24]. Among the KMTs screened, G9a exhibited the most significant expression changes, leading us to hypothesize that G9a-mediated regulation of H3K9 methylation is a key factor influencing HNSCC radiosensitivity.

Our study provides substantial evidence supporting the novel role of G9a in modulating radiosensitivity in HNSCC. Genetic or pharmacological inhibition of G9a significantly enhanced radiosensitivity both in vitro and in vivo. Notably, the epigenetic drug BRD4770, which inhibits G9a activity without affecting its expression, demonstrated potent efficacy as a radiosensitizer. While ionizing radiation (IR) typically induces apoptosis through caspase-mediated pathways [24–26], we found that G9a knockout did not significantly alter levels of γ-H2AX, caspase-3, or cleaved caspase-3, suggesting that apoptosis is not the primary mode of cell death in G9a-deficient cells. Instead, transmission electron microscopy revealed mitochondrial atrophy and increased membrane density - hallmark features of ferroptosis—in G9a knockout cells following IR. RNA sequencing and KEGG pathway analysis further confirmed that ferroptosis is a major cell death mechanism induced by G9a deficiency during radiotherapy.

Ferroptosis, a unique form of iron-dependent cell death driven by lipid peroxidation, is distinct from apoptosis, necrosis, and other cell death pathways [27]. It has been implicated in various cancer therapies, including radiotherapy, immunotherapy, and chemotherapy [8, 27, 28]. IR is a potent inducer of ferroptosis, often surpassing the efficacy of conventional ferroptosis inducers (FINs) [8]. However, cancer cells can develop resistance by upregulating ferroptosis defense mechanisms, such as SLC7A11 and GPX4, to promote survival during radiotherapy. In our study, G9a inhibition enhanced ferroptosis, increasing the susceptibility of HNSCC cells and xenograft tumors to IR. Mechanistically, we demonstrated that G9a regulates the expression of TMEM27 by modulating H3K9me2 levels at the TMEM27 promoter.

TMEM27, also known as transmembrane protein 27, is a glycoprotein and homolog of angiotensin-converting enzyme 2 (ACE2). While its role in cancer remains poorly understood, TMEM27 has been implicated in renal cell carcinoma and shown to enhance iron-mediated cell death in liver cancer [29, 30]. In our study, radiotherapy induced the overexpression of TMEM27 in G9a-deficient HNSCC cells, leading to downregulation of key ferroptosis defense genes, including SLC7A11, GPX4, and FSP1. These findings suggest that TMEM27 acts as a critical mediator of G9a-dependent ferroptosis and radiosensitivity. It should be mentioned that this study was limited by the reversal experiment, in which suppressing the levels of TMEM27 in G9a knockout cells should restore cellular radioresistance.

In summary, our study identifies G9a as a key epigenetic regulator of histone H3K9 methylation in HNSCC. Depletion of G9a enhances radiosensitivity by activating ferroptosis through the transcriptional regulation of TMEM27. Specifically, G9a binds to the H3K9me2 site at the TMEM27 promoter, silencing its expression. These findings provide valuable insights into the mechanisms underlying radioresistance in HNSCC and highlight the therapeutic potential of targeting the G9a- TMEM27-ferroptosis axis to improve radiotherapy outcomes.

## Materials and methods

### Reagents

BRD4770 was purchased from MCE (MedChemExpress, Shanghai, China), with an initial concentration of 10 mM (10 mg BRD4770 + 2.4186 ml DMSO). The BRD4770 was dissolved directly in 0.5% methylcellulose preheated at 37 °C, with a working solution concentration of 2 mg/ml for intraperitoneal injection in nude mice. Ferrostatin-1 (S7243), Z-VAD-fmk (S7023) and necrostatin-1s (S8037) were obtained from Selleck Chemicals (Houston, TX, USA).

### Cell culture

As described previously [31], the human HNSCC cell line Tu686 was kindly provided by Dr Zhuo G. Chen (Emory University, USA). The Fadu and SCC4 cell lines were purchased from ATCC. HN8 and JHU011 were kindly gifted by Dr Joseph Califano (University of California San Diego, USA). The precancerous oral mucosa cell line DOK was obtained from the Cell Bank of Type Culture Collection of the Chinese Academy of Sciences. These cells were cultured in appropriate growth medium supplemented with 10% fetal bovine serum and 1% penicillin‒streptomycin (Gibco, New York, NY, USA) in a humidified atmosphere (37 °C, 5% CO_2_). All experiments were conducted with mycoplasma-free cells.

### Patient samples

All patients’ tissue samples and information were obtained as our previous study [31]. The study was conducted in accordance with the Declaration of Helsinki and approved by the Research Ethics Committee of Xiangya Hospital, Central South University, and written informed consents were obtained from all these patients. The TNM stage was determined according to the 7th American Joint Committee on Cancer (AJCC) grading system.

### Irradiation

Irradiation was delivered at room temperature using an X-RAD 225 cabinet irradiator (Precision X-Ray, Madison, CT, USA) at a dose rate of 250 cGy/min. Compensation glue (0.5 cm thick) was used to cover the cell culture containers or mice. The source-to-skin distance was 40 cm.

### qRT-PCR

Total RNA was extracted from cell lines using TRIzol reagent (Invitrogen, Carlsbad, CA, USA). Total RNA was digested by RNase-free DNase I (Biosharp, Beijing, China). Synthesis of cDNA was performed using 2 μg total RNA from each sample using Reverse transcription Kit (Yeasen Biotech, Shanghai, China). Quantitative PCR (qPCR) was carried out using the Hieff qPCR SYBR Green Master Mix (Yeasen Biotech) and analyzed on the ABI QuantStudio 7 Flex Real-Time PCR System. Relative gene expression values were calculated using the 2^−△△CT^ method and normalized to that of β-actin. The sequences of the primers used are listed in the Supporting Information: Table S1.

### Western blot (WB)

Total protein was extracted by using ice-cold RIPA lysis buffer (NCM Biotech, Suzhou, China) with protease inhibitor cocktail and phosphatase inhibitor for 30–60 min, sonicated every 5 min and centrifuged at 12,000 × *g* for 10 min at 4 °C. The supernatant was collected and protein concentration was measured by BCA protein assay kit (Beyotime, Shanghai, China). 16–30 μg protein was loaded and electrophoresed on 8-12% SDS-PAGE gels, then transferred to a PVDF membranes (Millipore, Bedford, MA, USA). After sealing in NcmBlot blocking buffer (NCM Biotech) for 15 min, the membrane was incubated within primary antibody overnight at 4 °C. Then a horseradish peroxidase-conjugated secondary antibody (1:2000, Cell Signaling Technology, Danvers, MA, USA) was used. Target protein bands were visualized using super sensitive ECL luminescence reagent (Meilun Biotechnology Co, Ltd., Dalian, China) in a Chemiluminescence and Fluorescence Imaging System (Azure Biosystems C500, USA). The antibodies used in this study are listed in the Supporting Information: Table S2.

### Immunofluorescence staining

HNSCC cell lines from different treatment groups were grown on glass coverslips (12 mm) in 24-well plates. Slide cells were fixed with 4% paraformaldehyde for 20 min at room temperature and incubated with 0.3% Triton X-100 (Beyotime, ST797) to increase membrane permeability. After treatment with blocking solution, the cell slides were incubated with a rabbit monoclonal anti-human primary antibody against E-cadherin or vimentin at 4 °C overnight. After 3 washes with PBS, the cells were incubated in a dark box with Alexa Fluo 488 goat anti-rabbit IgG (H + L) and Alexa Fluor 594 goat anti-rabbit IgG (Jackson ImmunoResearch, West Grove, PA, USA). The cells’ nuclei were counterstained with DAPI (Sigma, St. Louis, MO, USA) for 5 min at room temperature and washed with a staining buffer. The coverslips were mounted onto glass slides with an anti-fade solution. The cells were imaged on a Zeiss upright fluorescence microscope (Oberkochen, Germany). Each group was observed under the same exposure conditions, such as the same fluorescence channel and the same laser intensity.

### Colony formation assays

Cells were serially diluted to appropriate concentrations, plated into 60-mm dishes in triplicate and allowed to attach for 12 h. Use a gradient of radiation doses (0, 2, 4, 6, and 8 Gy) to treat pores seeded with different cell densities, with three replicate wells per sample. Surviving colonies were stained with crystal violet 14 days later, and colonies larger than ~50 cells were counted. The surviving clonogenic fraction of the irradiated cells was normalized to the plating efficiency of the nonirradiated controls. The data are presented as the means ± SDs. The curve SF = 1-(1-e^-D/D0^)^N^ was fitted to the experimental data with GraphPad Prism 8.

### CCK8 and IC50

The logarithmic growth stage cells were diluted after digestion and inoculated into a 96-well plate. A pipette was used to add cells to each well, with 2 × 10^3^ cells per well. After 6 h of cell attachment, different concentrations of BRD4770 were added, and the cells were further cultured for 72 h. The drug concentrations used were 0, 1, 2, 4, 8, 16, 32, 64, 128, and 256 μM. Cell proliferation was measured using a CCK-8 kit (Beyotime). The indicated cells were seeded into 96-well plates at a density of 2 × 10^3^ cells per well in 100 μl complete medium. At different time points, medium was removed, 10 μl of CCK8 dye diluted in 100 μl DMEM was added. The spectrometric absorbance at the wavelengths of 450 nm was determined with a microplate reader (Biotek, Winooski, VT, USA). The assay was performed three times in triplicate. The curve was fit in GraphPad Prism 8 software, and the IC50 value was calculated.

### IHC

Immunohistochemical staining was performed as we described previously [31]. The tissue slides were stained with a diluted primary antibody against G9a (1:300, CST, USA) or Ki-67 (1:2000, Proteintech, China). The staining intensity of G9a was scored as 0 (no intensity), 1 (weak intensity), 2 (moderate intensity) or 3 (strong intensity). The degree of staining was scored as 0 (0%), 1 (1–25%), 2 (26–50%), 3 (51–75%) or 4 (76–100%) according to the proportion of immunopositive cancer cells. A histoscore of G9a was generated by the sum of the staining intensity and degree scores (overall score range, 0–7). Patients with HNSCC were classified into high expression (4–7) and low expression (0–3) groups based on the histoscore.

### Transfection

Fadu and HN8 cells were transfected with CRISPR/CAS9 single-vector lentivirus (Genechem, Shanghai, China). The sg sequences were as follows: LV-EHMT2-sgRNA-11418-1 (sg1): TCAGATTCATCCCCAATGAG; LV-EHMT2-sgRNA-11419-2 (sg2): CGGGCCAAGATGTCAATGAC; LV-EHMT2-sgRNA-11418-3 (sg3): GTAGCCTCATAGCCAAACTC; control empty Lv-vector-GV554 (sgCtrl). Lv-hG9a-79759 and Lv-hG9a with G9a-ΔSET lentiviruses (Genechem) were used to transfect sg1 Fadu cells and sg1 HN8 cells (sg1-hG9a, sg1-hG9a-ΔSET), and the empty vector Lv-GV341 (sg1EV) was used as the control.

### Generation and validation of CRISPR knockout cell lines

After the cells that were transfected with the lentiviruses were digested, 10 ml of complete culture medium with a total of ~100 cells was prepared, the mixture was placed in a transfer tank, and 96-well plates were seeded. After 24 h, the cells in each well of the 96-well plate were observed under a fluorescence microscope, and one cell with fluorescence in each well was selected and labeled. The medium was changed the next day, and the samples were passaged ~1 month later to complete amplification. Monoclonal cells were collected, proteins were extracted, knockout status was verified by Western blot, and Sanger sequencing was performed to determine whether the knockout was homozygous or heterozygous.

### Sanger sequencing

The DNA was extracted with a DNA extraction kit, the concentration and purity of the DNA were measured using a spectrophotometer, and the OD 260/280 was detected within the range of 1.7–2.0. PCR amplification of DNA was performed according to Yeasen ‘s instructions (Yeasen Biotechnology), and Primer Premier 5 software was used to design the following primers: sgRNA-F5′-GACACCCCTCGTAGTGAAGAAAC-3′ and sgRNA-R5′-CTCCCATCCCACTCACCTGTC-3′. The PCR products were analyzed by 1% agarose gel electrophoresis. According to the instructions provided with the SanPrep column DNA gel recovery kit (Sangon Biotech, Shanghai, China), the PCR strips were collected and cut into pieces. We then performed machine sequencing, searched for results in the result group, and analyzed the results using sequence analysis software. Sequence alignment was performed using SeqMan software.

### Transmission electron microscopic examination

One milliliter of sample was fixed for electron microscopic evaluation. The sample was placed at 4 °C for delivery to the TEM laboratory at the Pathology Department of Xiangya Hospital, Changsha, Hunan. The samples were washed three times with Millonig phosphate buffer at 10 min intervals, incubated in 1% osmium tetroxide for 1 h and washed three times with Millonig phosphate buffer at 10 min intervals. The sample was then dehydrated at room temperature in a graded series of 50%, 70%, and 90% acetone, with 10 min intervals between each step, and then twice with 100% acetone at 15 min intervals. The sample was then immersion embedded in resin by first soaking the sample in a 1:1 mixture of acetone and resin for 12 h and then polymerizing it in 100% resin overnight at 37 °C. The sample resin curing process involved polymerizing the sample in 100% resin overnight at 37 °C and then polymerizing the sample at 60 °C for 12 h. After double staining with 3% uranyl acetate and lead nitrate, the samples were examined and photographed under a Hitachi HT-7700 electron microscope.

### RNA-seq

Briefly, total RNA was extracted using the TRI reagent (Sigma-Aldrich), and the quality of the extracted total RNA samples was examined with RNA 6000 Nano Kit (Agilent, Santa Clara, CA, USA). The MGIEasy RNA Library Prep Kit (MGI Tech, Shenzhen, China) was used for library preparation according to the manufacturer’s instructions. Briefly, after mRNA enrichment, the samples were incubated with fragmentation buffer to obtain a target insert fragment size of ~150 bp. Then, the fragments were reverse-transcribed into cDNA. After repair and A-tailing, the double-stranded cDNA products were ligated with adapters and subjected to PCR amplification (95 °C for 30 s, 56 °C for 30 s, and 72 °C for 60 s; 14 cycles). The PCR products were cleaned using DNA Clean Beads. Then, quality control of the purified PCR products was carried out using a DNA 1000 Kit (Agilent). PCR products at ~230 bp in size were subjected to multiple-sample pooling, amplification, and digestion to obtain the libraries. Gene expression analysis was performed by mRNA sequencing on a BGISEQ-500 instrument (MGI Tech) with a single-end 50 bp module. The raw RNA-seq files have been uploaded to the NCBI database (No. PRJNA1153734).

### Lipid peroxidation assay

The C11-BODIPY probe (Thermo Scientific, Waltham, MA, USA) is a lipid-soluble reactive oxygen species that can penetrate the cell membrane to detect lipid peroxidation in the cell membrane and within the cell. Cells were seeded in a 6-well plate at a density of ~50%. The cells from the different treatment groups were added to 5 μM BODIPY serum-free medium, incubated in an incubator for 30 min, digested with trypsin, and centrifuged at 1000 rpm for 5 min. The cells were washed with PBS containing 2% serum twice, centrifuged at 1000 rpm, and incubated for 5 min. The cells were resuspended in PBS containing 2% serum and stored at 4 °C in the dark, and flow cytometry was used to detect the ROS levels.

### Chromatin immunoprecipitation (ChIP)

ChIP analysis was performed as described in the SimpleChIP® Enzymatic Reagent Kit (Cell Signaling Technology). Briefly, a 10 cm plate with a cell count of 4 × 10^6^ cells was crosslinked with 1% formaldehyde for 10 min at room temperature, and the reaction was stopped with the addition of 125 mM glycine. The cell nuclei were prepared, and the chromatin was digested by enzymatic hydrolysis according to the instructions. Moderate ultrasonication (power 25 W, 20 s pulse, 3 groups) was used with the samples placed on ice during each pulse interval, and the purified DNA was observed on a 1% agarose gel to ensure that the DNA length was 150–900 bp. The mixture was centrifuged at 4 °C and 9400 rpm for 10 min, and the supernatant was collected to obtain the cross-linked chromatin. The DNA was purified in a DNA purification column. The diluted chromatin was added to antibody mixtures as follows: positive control, 10 μL of Histone H3 (D2B12) XP rabbit antibody was added; negative control, 2 μL of normal IgG rabbit antibody was added. Magnetic bead elution of chromatin, reverse cross-linking and DNA purification were then performed. Quantitative analysis was performed using qPCR, and the primer sequences used for performing ChIP analysis are listed in Table S1.

### Xenograft tumor model

HNSCC cell aliquots (100 μL) of 2.0 × 10^6^ cells were injected subcutaneously into 5-week-old male BALB/c nude mice. The mice were randomized into experimental groups when their tumors reached 80–100 mm^3^. The irradiation dose was 4 Gy once a day for a total of 2 consecutive days. A lead plate was used to cover other parts of the nude mouse during irradiation. The tumor volume was determined every 3 days with a calliper and calculated using the modified ellipse formula (volume = length × width^2^/2). When the tumor volume reached 1000 mm^3^, the mice were sacrificed. For the G9a inhibition experiments, BRD4770 was given 24 h prior to radiation as well as by intraperitoneal injection the next day. All the animal studies were conducted with the approval of the Committee on the Use and Care of Animals of Xiangya Hospital.

### TCGA data

RNA-sequencing expression (level 3) profiles and corresponding clinical information for patients with HNSCC were downloaded from the TCGA dataset (TCGA, https://portal.gdc.cancer.gov/). Statistical analyses were performed using R software v4.0.3 (R Foundation for Statistical Computing, Vienna, Austria). One-way analysis of variance (ANOVA) was conducted, and genes were considered differentially expressed when the log2-fold change cut-off value was 1 and the p value cut-off was 0.01.

### Statistical analysis

The data are shown as the representative results of at least three independent experiments. Statistical analyses of two groups were performed with the unpaired Student’s t test (for equal variances) or the Mann-Whitney U test (for unequal variances) using SPSS software (version 23.0; SPSS, Inc., Chicago, IL, USA). The chi-square test was used for statistical analysis of categorical data. P < 0.05 was considered statistically significant.

## Supplementary information

Table S1

Table S2

Figure S1

Figure S2

Figure S3

Figure S4

Figure S5

Figure S6

Supplementary Figure and Table Legends

Original data of western blots

## Data Availability

Our sequencing data can be downloaded from the NCBI database (No. PRJNA1153734). The data supporting the findings of this study are available from the corresponding author upon reasonable request.
