# Supplementary material for: G9a deficiency activates TMEM27 to promote ferroptosis and enhances radiosensitivity in head and neck squamous cell carcinoma
Source: Cell Death Discov. 2025 Nov 10;11:517. doi: 10.1038/s41420-025-02805-1 (PMC12603116; doi:10.1038/s41420-025-02805-1)
Supplement: Supplementary file 1 — Table S1 [file 41420_2025_2805_MOESM1_ESM.docx]

**Table S1. The sequence of qRT-PCR primers**

| Gene name | Primer sequence (5’-3’) |
| --- | --- |
| SUV39H1 |  |
| Forward | AGGAGCTCACCTTTGATTACAA |
| Reverse | GTCCCACACTTGCATTCAATAC |
| SUV39H2 |  |
| Forward | ATTGATAACCTCGATACTCGTCTT |
| Reverse | TCTCCAGAACCTTTCATTTGATAA |
| KMT1D |  |
| Forward | AAAGAACAAGGAAGGAGAGACG |
| Reverse | AAAATGCTCTCCTTAGAAGGCT |
| SETDB1 |  |
| Forward | GGGCAAGGGTGTTTTCATTAAC |
| Reverse | GTTAGTTGATGGCAGGCACACTT |
| KDM3A |  |
| Forward | AAGGTGTGTGTGGAATTTGATG |
| Reverse | AAAATGCTCTCCTTAGAAGGCT |
| KDM3B |  |
| Forward | TATGGCACATCTATGCAGCC |
| Reverse | GAATTGGGTCATGATCAGGG |
| KDM4A |  |
| Forward | GTGGTCTTCATTACCTGCTTTC |
| Reverse | TTGACTTCATAGAAGGTCTCGG |
| KDM4B |  |
| Forward | AAGACTTCAACAAATACGTGGC |
| Reverse | TGGATATTGTACTGCGTGAAGA |
| KDM4C |  |
| Forward | GCAGCAATGAAGAAAATGATGC |
| Reverse | AAACACATCTCTGGTATGAGGG |
| KDM4D |  |
| Forward | GGGCAGGGGTGTTTACTCAAT |
| Reverse | TGTTTGCCAAATGGCGATACT |
| JMJD1C |  |
| Forward | CAGGTCTCGTGCCAATCAAAA |
| Reverse | GCTGTTGCTGGTGTGTATTCT |
| GAPDH | |
| Forward | TCCAAAATCAAGTGGGGCGA |
| Reverse | AGTAGAGGCAGGGATGATGT |
| β-actin | |
| Forward | TGGCACCCAGCACAATGAA |
| Reverse | CTAAGTCATAGTCCGCCTAGAAGCA |
| TMEM27 promoter1 | |
| Forward | GTTTGTTTCCTGAATGAAGATAGCC |
| Reverse | GTTTTCCCTACTCTGACTCTACCTC |
| TMEM27 promoter2 | |
| Forward | GGTGACTGCCATTCATGCTG |
| Reverse | CACTCACTGCCCCAACTTTC |
