# Supplementary material for: G9a deficiency activates TMEM27 to promote ferroptosis and enhances radiosensitivity in head and neck squamous cell carcinoma
Source: Cell Death Discov. 2025 Nov 10;11:517. doi: 10.1038/s41420-025-02805-1 (PMC12603116; doi:10.1038/s41420-025-02805-1)
Supplement: Supplementary file 2 — Table S2 [file 41420_2025_2805_MOESM2_ESM.docx]

**Table S2. The antibody information**

| Mono-Methyl-HistoneH3(Lys9) (D1P5R) Rabbit | 1:1000 dilution，Cell Signaling Technology, Danvers, MA |
| --- | --- |
| Di-Methyl-HistoneH3(Lys9)(D85B4) XP® Rabbit | 1:1000 dilution, Cell Signaling Technology, Danvers, MA |
| Tri-Methyl-Histone H3 (Lys9) (D4W1U) Rabbit | 1:1000 dilution, Abcam(ab1416), Cambridge, UK |
| G9a/EHMT2 (D5R4R) XP® Rabbit | 1:1000 dilution, Cell Signaling Technology, Danvers, MA |
| SETDB1/EHMT1 (E6Q8B) Rabbit | 1:1000 dilution, Cell Signaling Technology, Danvers, MA |
| SUV39H1 (D11B6) Rabbit | 1:1000 dilution, Cell Signaling Technology, Danvers, MA |
| SUV39H2 Polyclonal antibody | 1:1000 dilution, Cell Signaling Technology, Danvers, MA |
| Caspase-3 Antibody | 1:1000 dilution, Cell Signaling Technology, Danvers, MA |
| Cleaved Caspase-3 (Asp175) (5A1E) Rabbit | 1:1000 dilution, Cell Signaling Technology, Danvers, MA |
| SLC7A11/xCT Polyclonal antibody | 1:10000 dilution, Proteintech, Wuhan, China |
| GPX4 (E5Y8K) Rabbit | 1:1000 dilution, Cell Signaling Technology, Danvers, MA |
| AIFM2/FSP1 Antibody | 1:1000 dilution, Cell Signaling Technology, Danvers, MA |
| DHODH Polyclonal antibody | 1:10000 dilution, Proteintech, Wuhan, China |
| Anti-Tmem27/CLTRN antibody | 1:1000 dilution, Abcam(ab1416), Cambridge, UK |
| Vinculin Mouse Antibody | 1:2000 dilution, Affinity, Jiangsu, China |
| α-Tubulin | 1:5000 dilution, ABclonal Technology, Wuhan, China |
| GAPDH | 1:10000 dilution, ABclonal Technology, Wuhan, China |
| β-Actin (8H10D10) Mouse | 1:1000 dilution, Cell Signaling Technology, Danvers, MA |
| Phospho-Histone H2A.X Rabbit | 1:1000 dilution, Cell Signaling Technology, Danvers, MA |
| Ki67 Antibody | 1:100 dilution, Affinity, Jiangsu, China |
| Anti-rabbit IgG | 1:2000 dilution, Cell Signaling Technology, Danvers, MA |
| Anti-mouse IgG | 1:1000 dilution, Cell Signaling Technology, Danvers, MA |
| Alexa Fluor 488 Rabbit IgG | 1:1000~1:2000 dilution, Jackson Immuno Research, West Grove, PA |
