# Supplementary figures and images for: G9a deficiency activates TMEM27 to promote ferroptosis and enhances radiosensitivity in head and neck squamous cell carcinoma
Source: Cell Death Discov. 2025 Nov 10;11:517. doi: 10.1038/s41420-025-02805-1 (PMC12603116; doi:10.1038/s41420-025-02805-1)

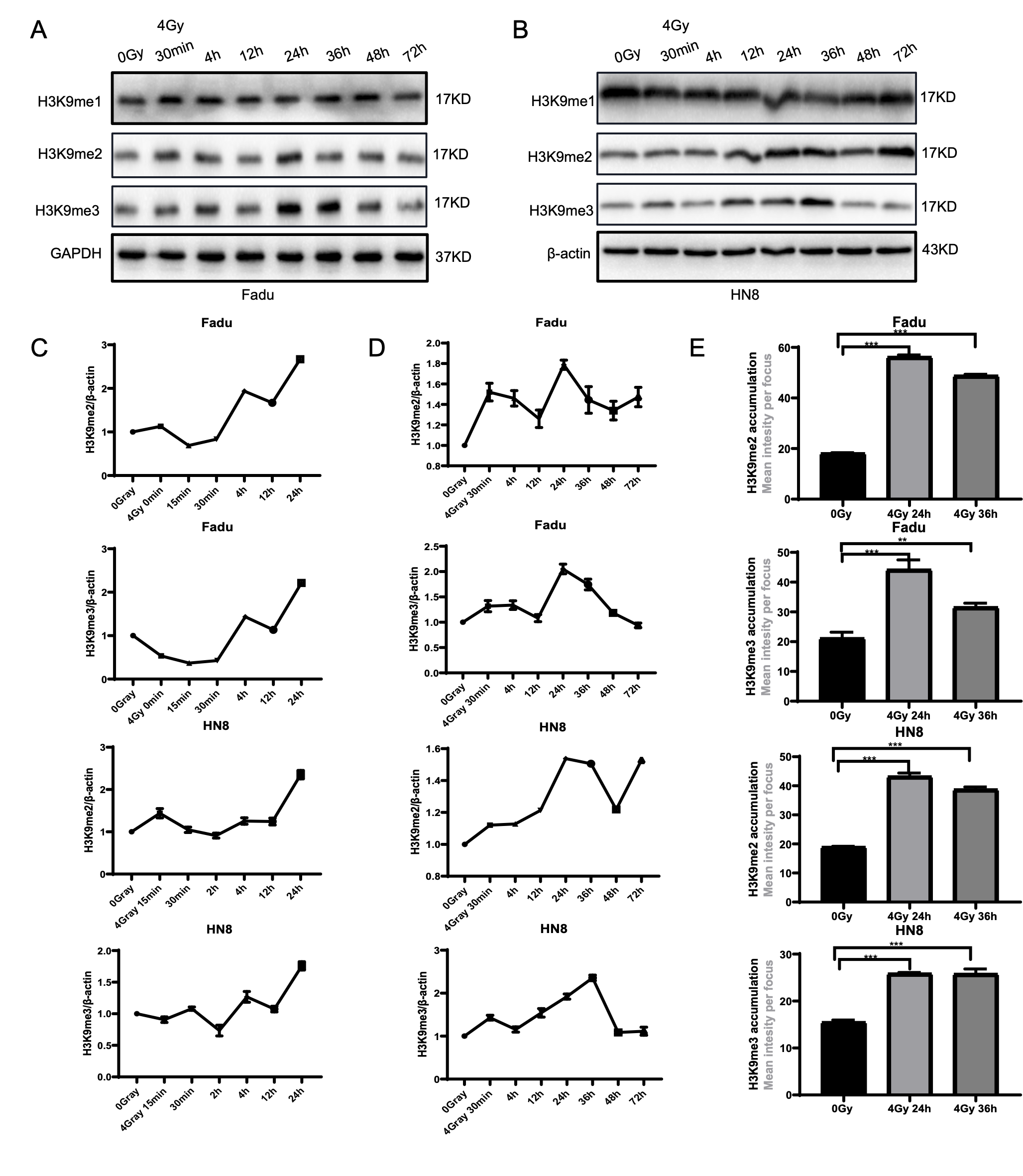

Supplement: Supplementary file 3 — Figure S1 [file 41420_2025_2805_MOESM3_ESM.png]

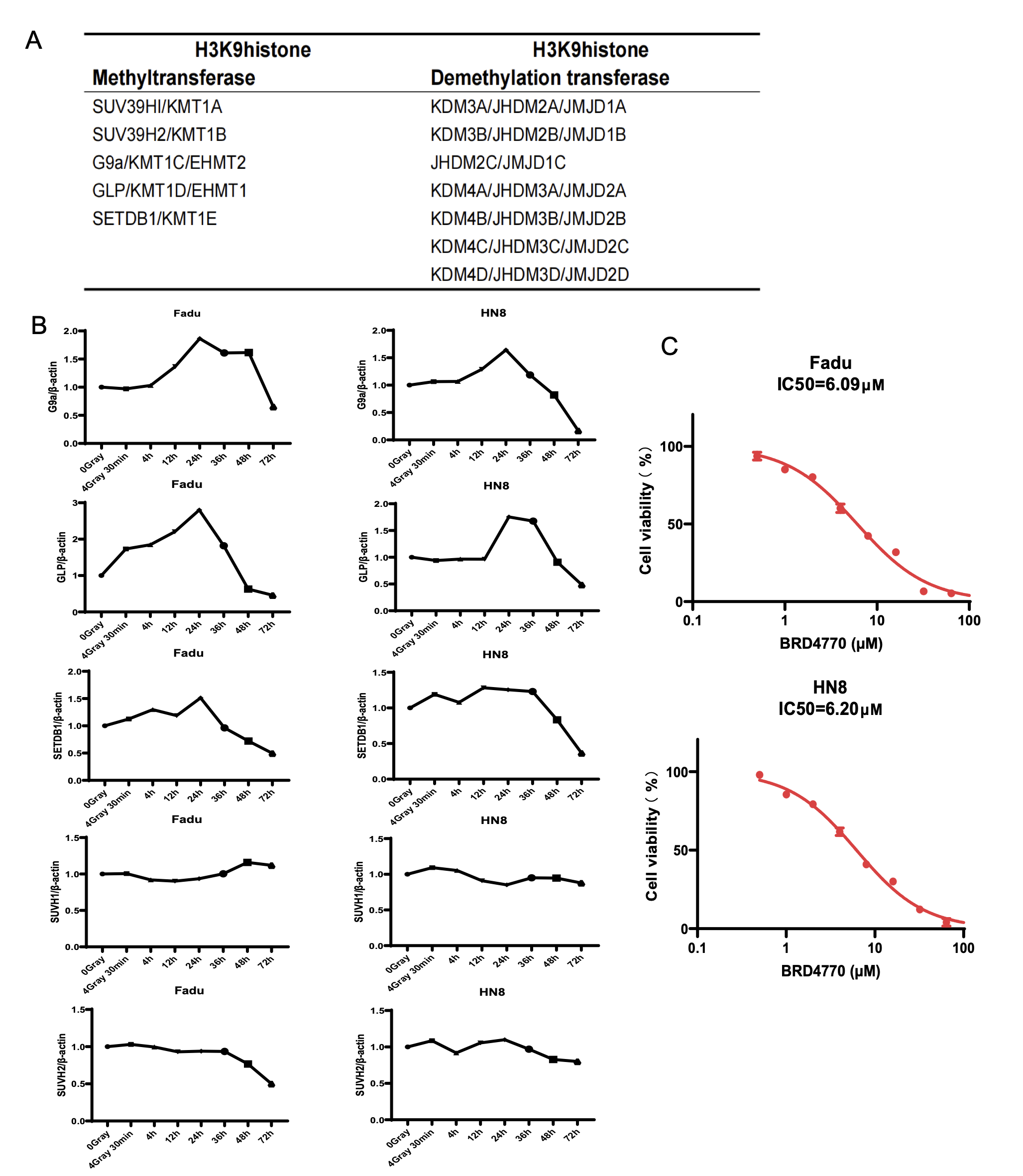

Supplement: Supplementary file 4 — Figure S2 [file 41420_2025_2805_MOESM4_ESM.png]

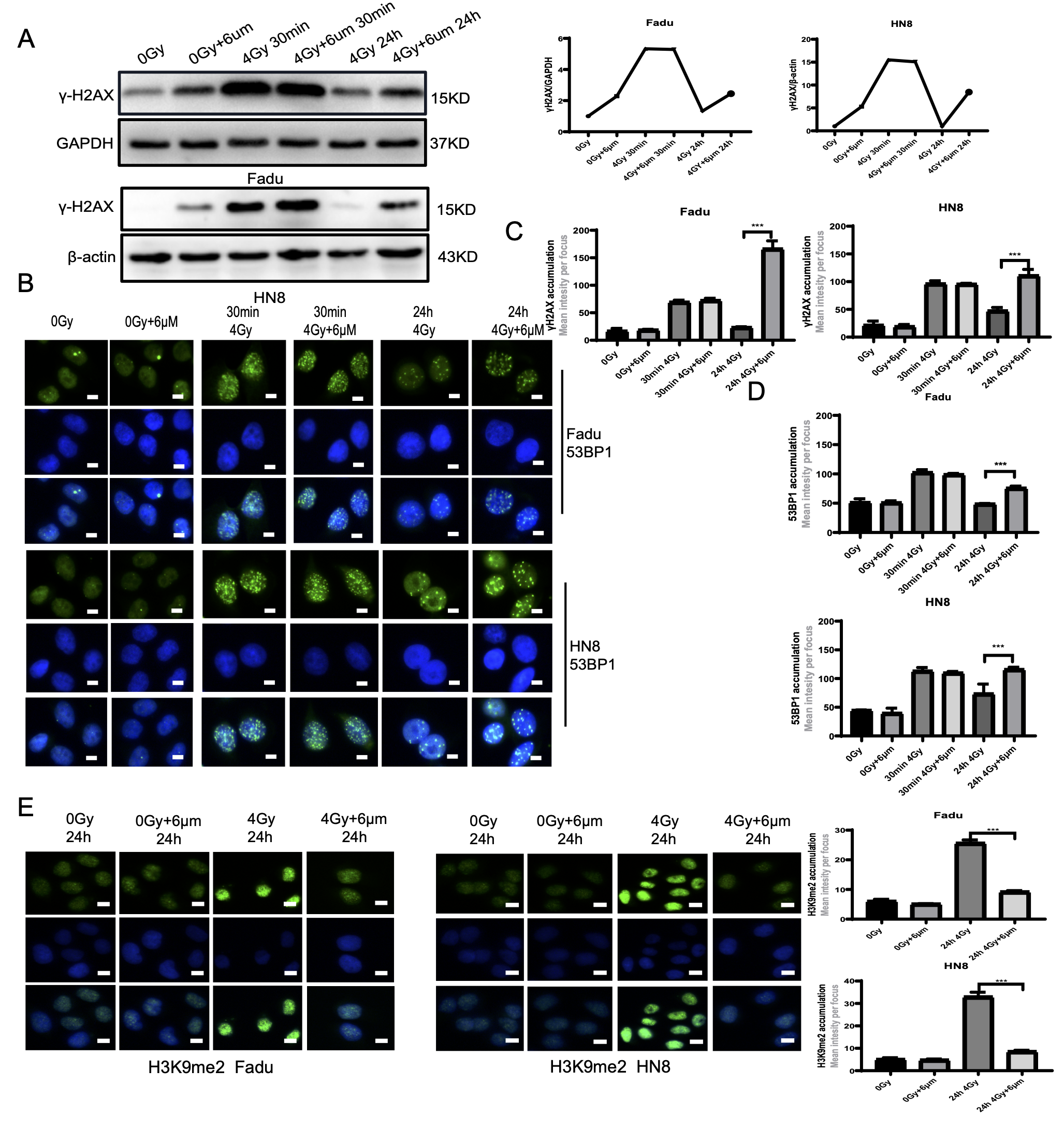

Supplement: Supplementary file 5 — Figure S3 [file 41420_2025_2805_MOESM5_ESM.png]

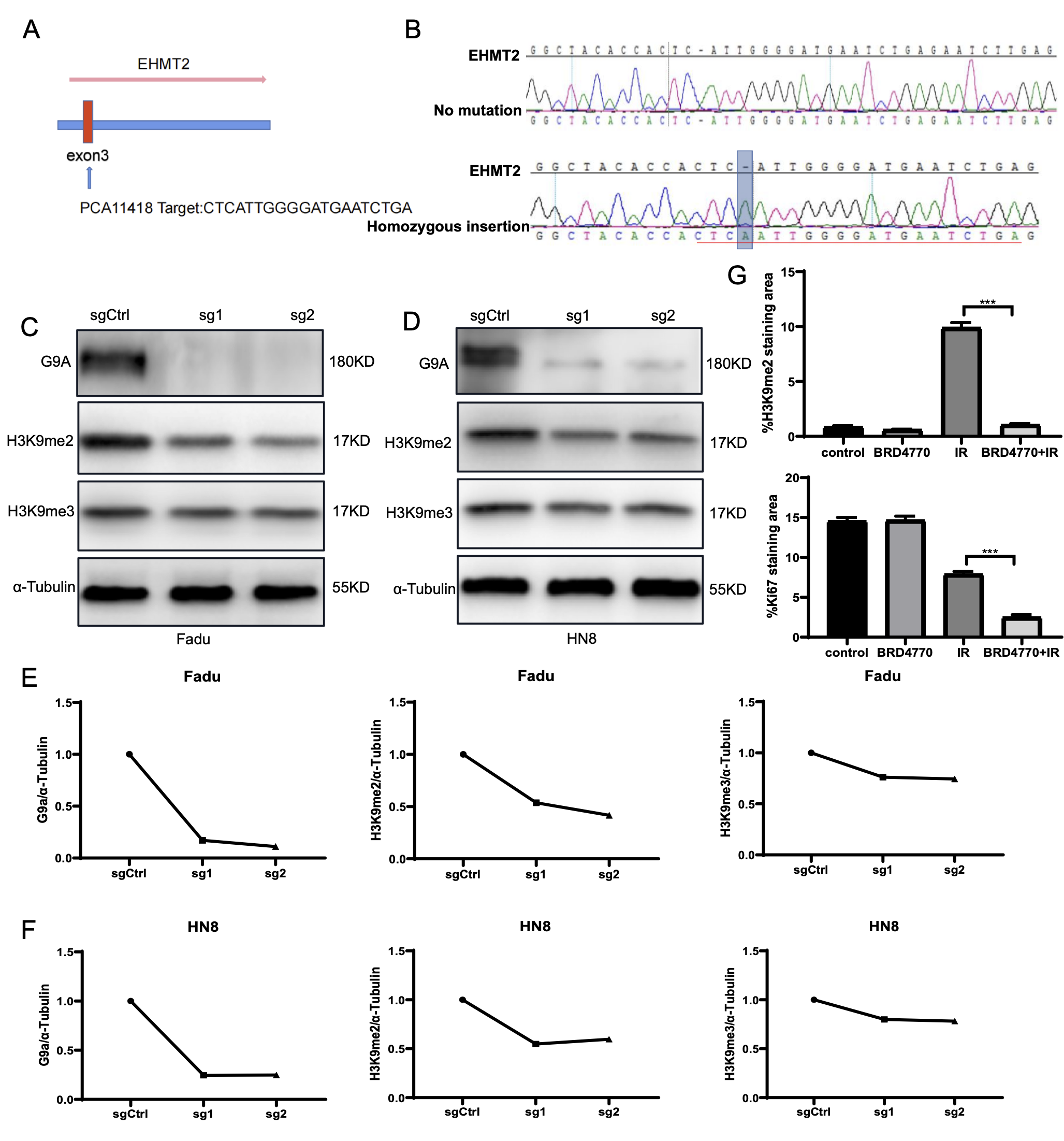

Supplement: Supplementary file 6 — Figure S4 [file 41420_2025_2805_MOESM6_ESM.png]

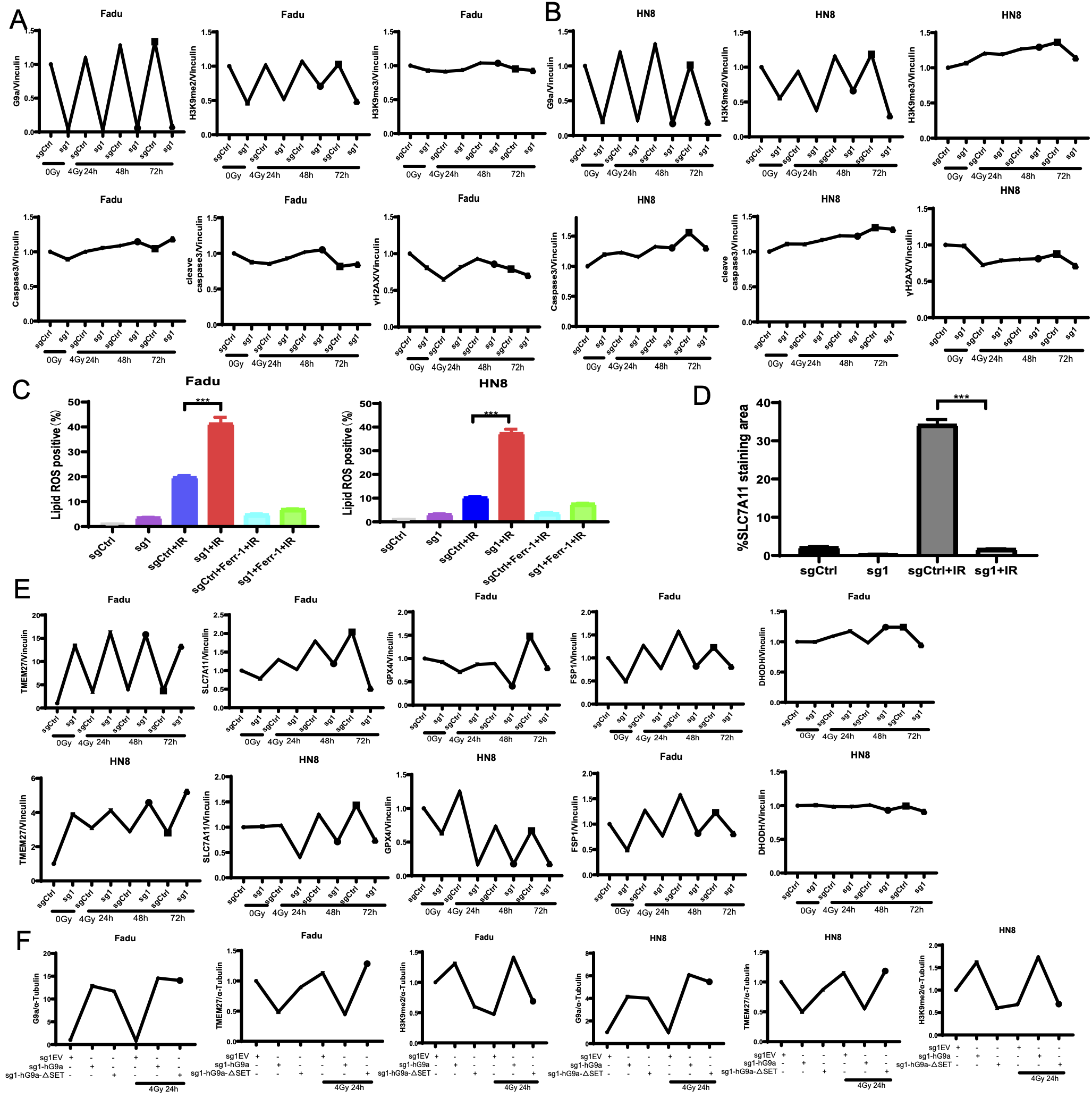

Supplement: Supplementary file 7 — Figure S5 [file 41420_2025_2805_MOESM7_ESM.png]

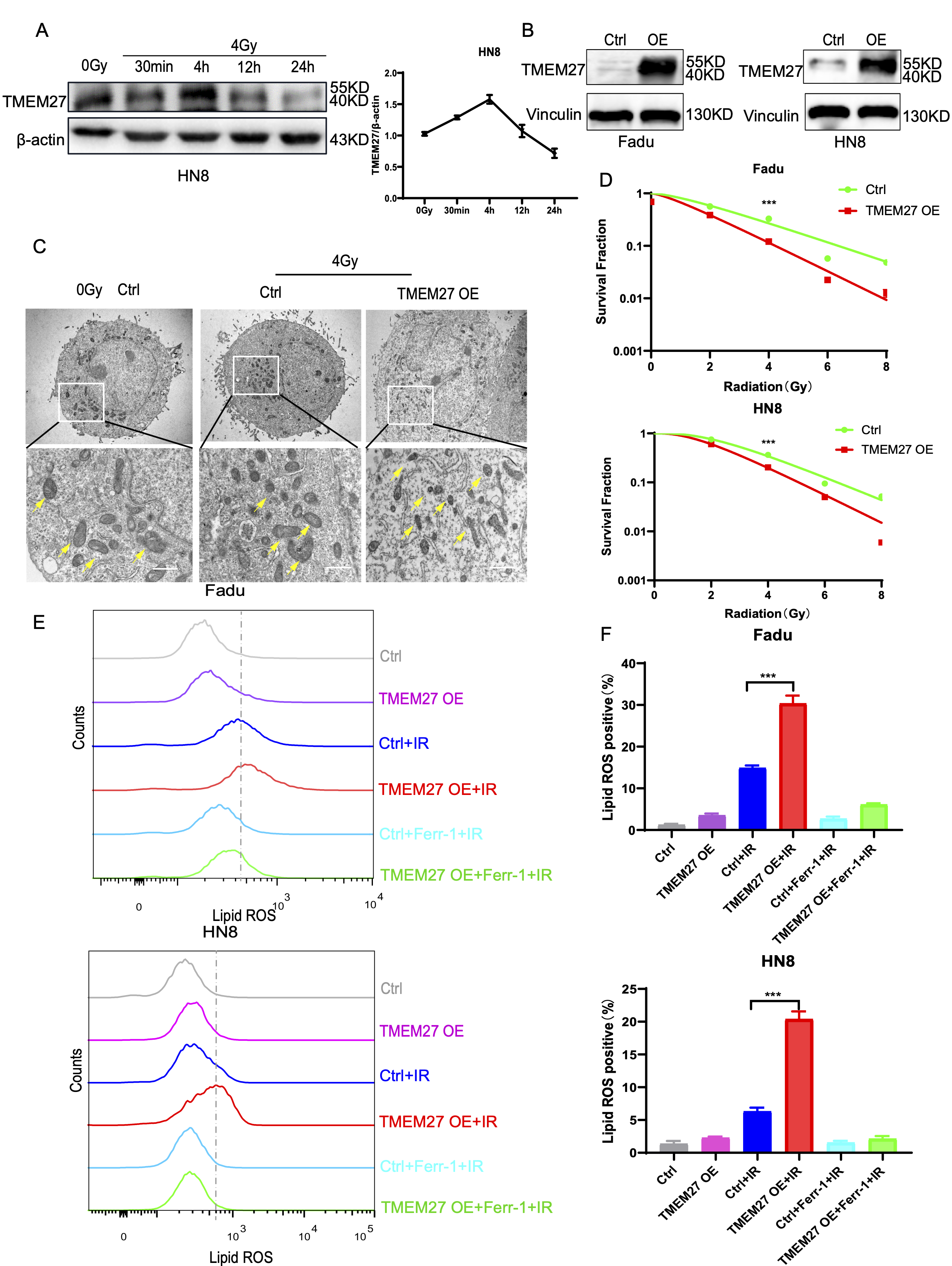

Supplement: Supplementary file 8 — Figure S6 [file 41420_2025_2805_MOESM8_ESM.png]
