# Supplementary material for: G9a deficiency activates TMEM27 to promote ferroptosis and enhances radiosensitivity in head and neck squamous cell carcinoma
Source: Cell Death Discov. 2025 Nov 10;11:517. doi: 10.1038/s41420-025-02805-1 (PMC12603116; doi:10.1038/s41420-025-02805-1)
Supplement: Supplementary file 9 — Supplementary Figure and Table Legends [file 41420_2025_2805_MOESM9_ESM.docx]

**Supplementary Figure Legends**

**Figure S1. Changes in the methylation site of histone H3K9 after incubation of HNSCC cells irradiated with 4 Gy for different durations.**

(A, B) After 4 Gy irradiation of Fadu or HN8 cells, the protein expression levels at H3K9me1 sites did not change, whereas the protein expression levels at H3K9me2 and H3K9me3 sites changed significantly. Within 72 h, the protein expression levels at H3K9me2 and H3K9me3 sites were highest at 24 hours in Fadu cells. (C, D) Trend line chart of H3K9me2 and H3K9me3 protein expression within 4 Gy irradiation 24 or 72 h in Fadu and HN8 cells are shown. (E) Quantitation of H3K9me2 and H3K9me3 in the nucleus of HNSCC cells after 4 Gy irradiation and incubation for 24 h and 36 h (Related to Fig. 1C, 1D).

**Figure S2. Presentation of KMTs and KDMs, the expression levels of H3K9 methyltransferase proteins after 4 Gy irradiation, and IC50 of BRD4770 in HNSCC.**

(A) Histone H3K9 lysine methyltransferases (KMTs) and demethyltransferases (KDMs). (B) Trend line chart of H3K9 methyltransferase proteins after 4 Gy irradiation in Fadu and HN8 cells (Related to Fig. 2C, 2D). (C) Dose response curves and IC50 determinations of Fadu and HN8 HNSCC cells to BRD4770.

**Figure S3. Effects of BRD4770 combined with radiotherapy on γ-H2AX, 53BP1 and H3K9me2 Foci.**

(A) Weatern blot showed the effects of BRD4770 combined with radiotherapy on DNA damage repair in Fadu and HN8 cells. (B) Fadu and HN8 cells were treated with 6 µM BRD4770 for 4 h, irradiated with 4 Gy, and fixed at 30 min and 24 h post-irradiation. Nuclei were stained with DAPI, and 53BP1 foci (green) were visualized to assess DNA damage; scale bar, 10 µm. (C，D) Quantification of γ-H2AX and 53BP1 expressions were detected using immunofluorescence staining. (E) Fadu cells and HN8 cells were treated with 6 µM BRD4770 for 4 h, irradiated with 4 Gy, and fixed for 24 h. DAPI was used to stain the nuclei; green indicates H3K9me2; scale bar, 10 µm.

**Figure S4. Validation of histone methyltransferase G9a knockout in HNSCC.**

(A) Using CRISPR-Cas9 genome editing technology, the sg1 sequence was inserted into the EHMT2 exon region. (B) Sanger sequencing revealed that the homozygous insertion of base A in the exon region of Fadu EHMT2 KO resulted in a homozygous deletion of EHMT2. (C, D) Western blot validation of the expression levels of the G9a, H3K9me2, and H3K9me3 proteins in Fadu EHMT2 KO and HN8 EHMT2 KO cells. (E, F) Trend line chart of G9a, H3K9me2 and H3K9me3 protein expression in Fadu EHMT2 KO and HN8 EHMT2 KO cells. (G) Immunohistochemical quantitative analysis of H3K9me2 and Ki67 expression levels in tumor tissues from treated mice in Fig. 3H.

**Figure S5. The quantitative analysis related to Figure 4, Figure 5 and Figure 6.**

(A, B) Trend line chart of the expression levels of G9a, H3K9me2, H3K9me3, γ-H2AX, and apoptosis-related proteins in Fig. 4E and 4F. (C) Lipid peroxidation levels of Fig. 5C were detected by flow cytometry after staining with 5 µM BODIPY-C11 probes. (D) Immunohistochemical quantitative analysis of SLC7A11 in tumor tissues from mice in Fig. 5F. (E) Trend line chart of the expression levels of TMEM27 protein levels and key proteins in the ferroptosis defense system in Fig. 6C. (F) Trend line chart of the expression levels of TMEM27 protein expression and ferroptosis defense system proteins in Fadu and HN8 cells in Fig. 6E.

**Figure S6. Overexpression of TMEM27 in Fadu and HN8 cells promotes ferroptosis and enhances radiotherapy sensitivity.**

1. Following 4 Gy irradiation of HN8 cells, Western blot showed the expression of TMEM27. (B) Western blot was used to validate the expression of TMEM27 in Fadu and HN8 overexpressed cells. (C) TEM images revealed the changes in mitochondrial morphology before and after radiotherapy in the TMEM27 overexpressed Fadu cells. (D) Fadu and HN8 cells with TMEM27 overexpression were irradiated with 0-8 Gy. Survival curves were generated using a multiple-target single-hit model, and two-way ANOVA was used to compare the survival curves between the Ctrl and OE groups (p < 0.001). (E, F) Changes of ROS levels were detected using the ferroptosis inhibitor Fer-1 and the C11-BODIPY probe in the TMEM27 overexpressed Fadu and HN8 cells after irradiation with 0 Gy and 4 Gy. Ctrl, Control; OE, Overexpression.

**Supplementary Table Legends**

**Table S1. The sequence of qRT-PCR primers**

**Table S2. The antibody information**
