# Supplementary material for: G9a deficiency activates TMEM27 to promote ferroptosis and enhances radiosensitivity in head and neck squamous cell carcinoma
Source: Cell Death Discov. 2025 Nov 10;11:517. doi: 10.1038/s41420-025-02805-1 (PMC12603116; doi:10.1038/s41420-025-02805-1)
Supplement: Supplementary file 10 — Original data of western blots [file 41420_2025_2805_MOESM10_ESM.pdf]

Figure1A:

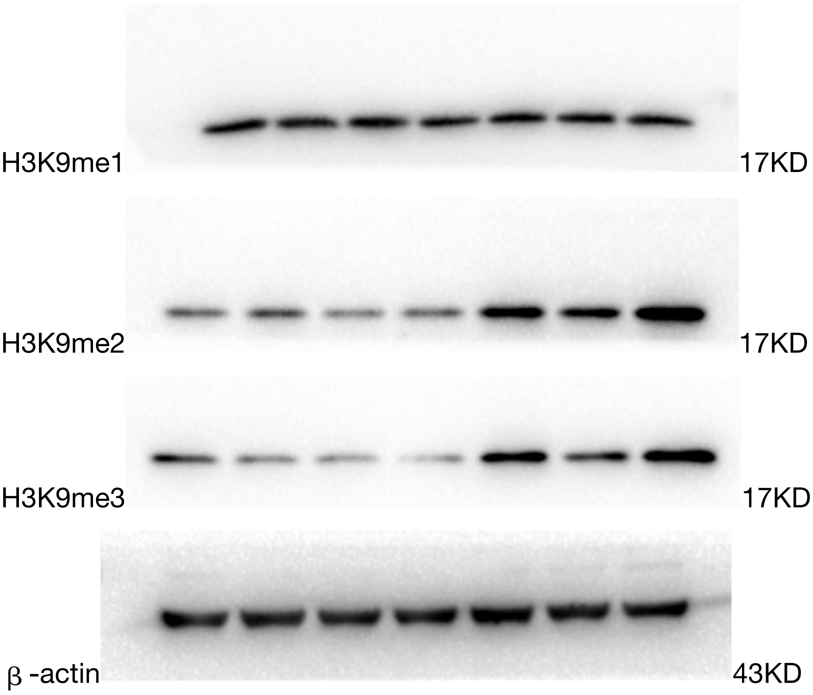

Figure1B:

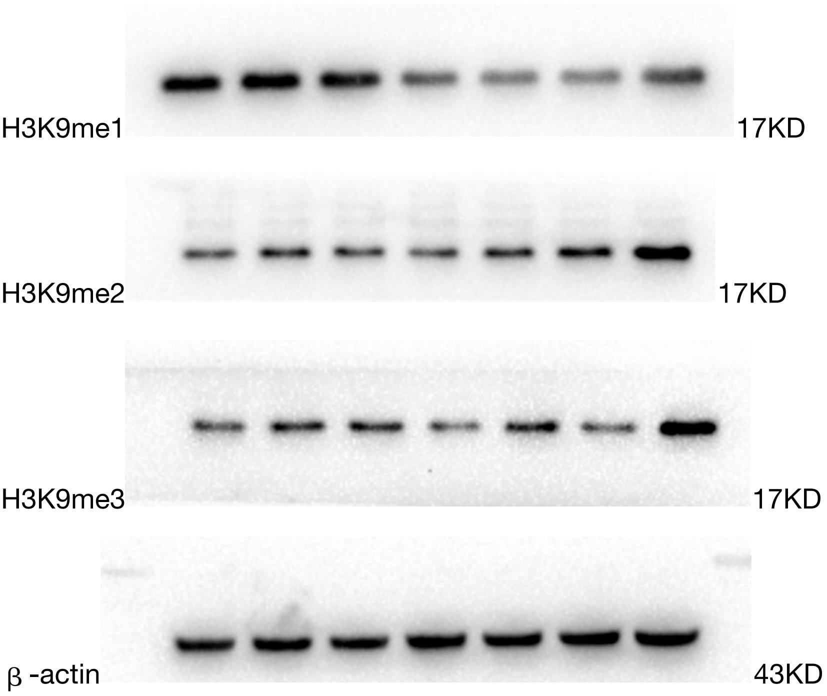

Figure2C Fadu

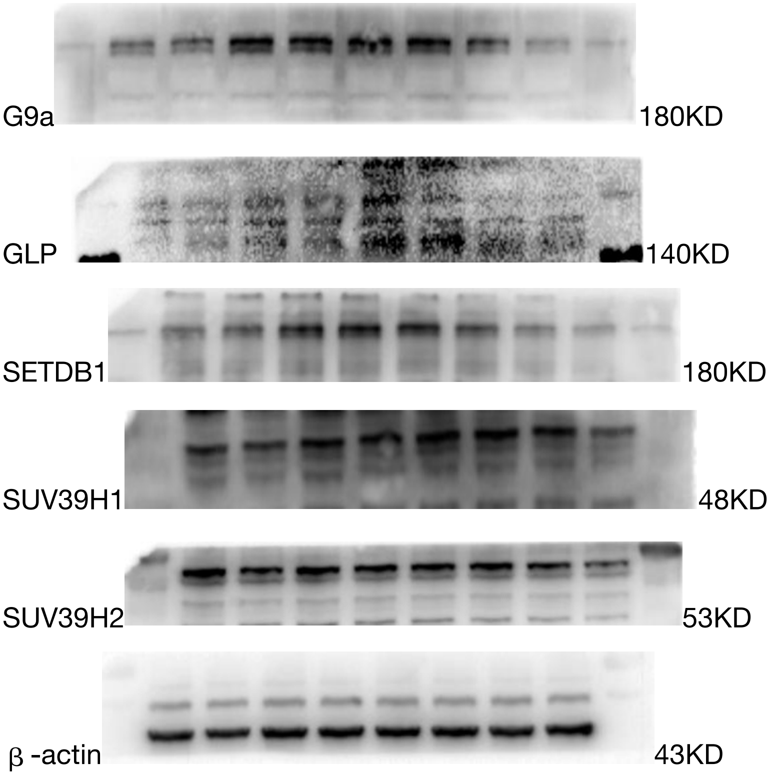

Figure2D HN8

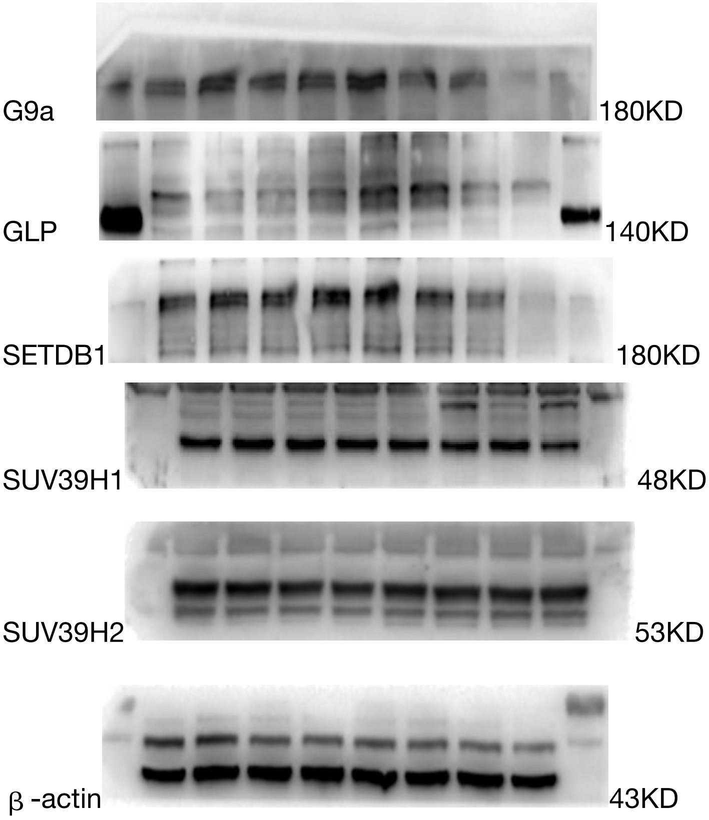

Figure4E

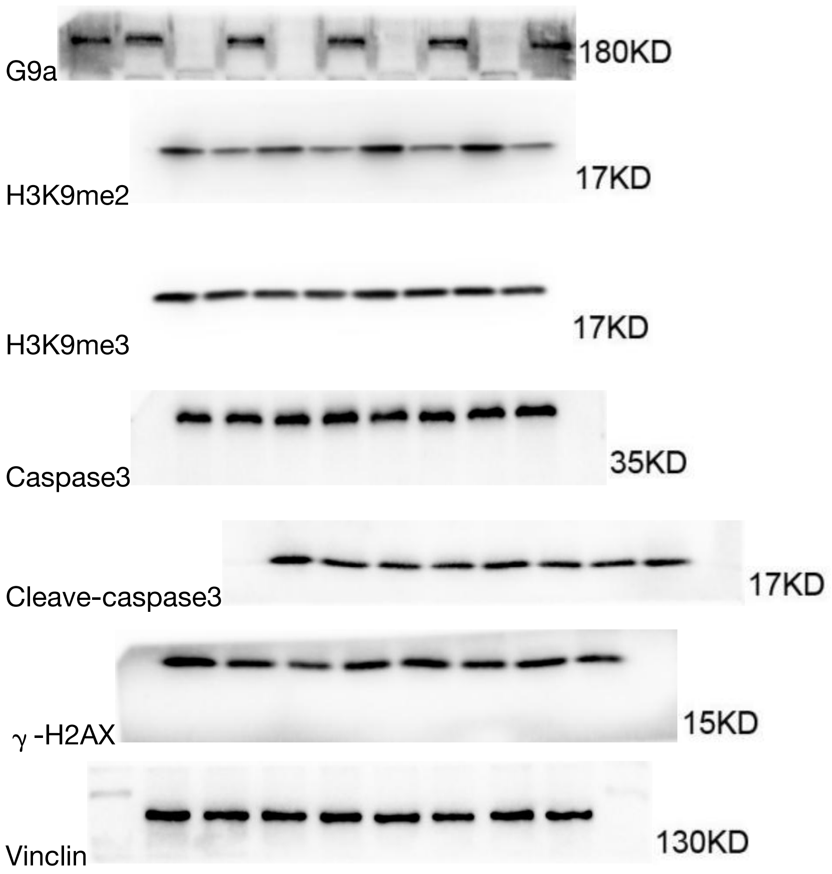

Figure4F

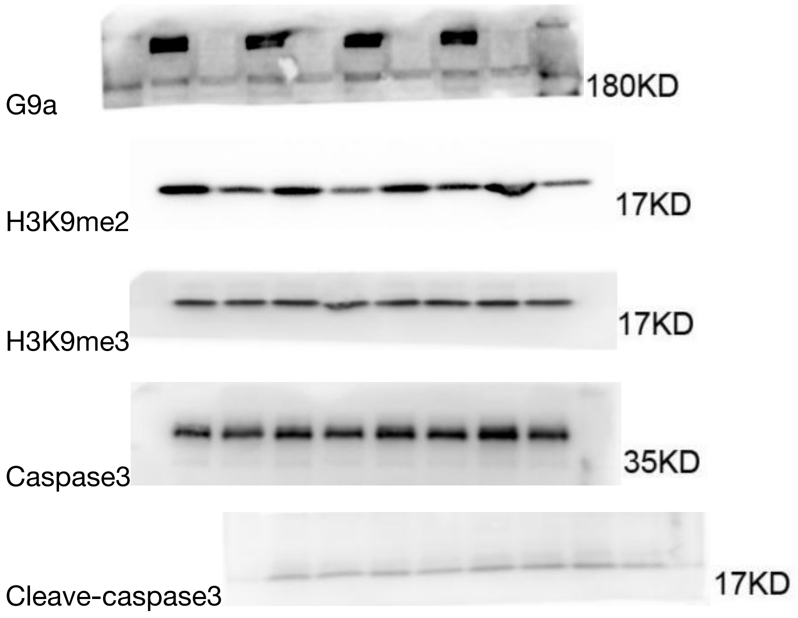

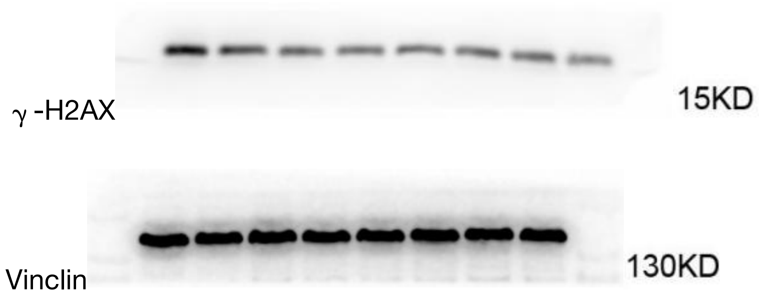

Figure6C Fadu:

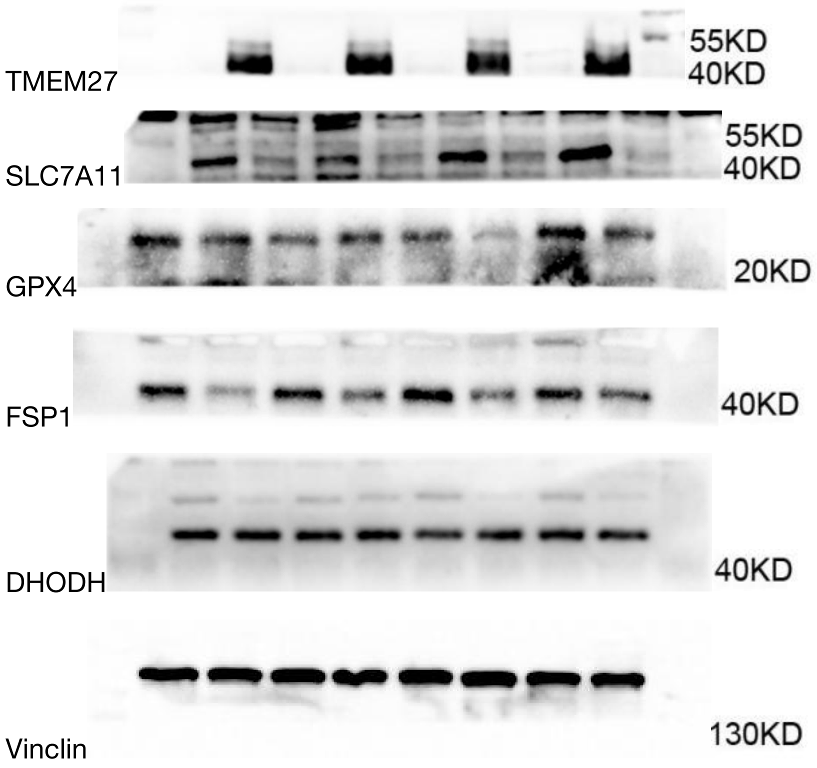

Figure6C HN8

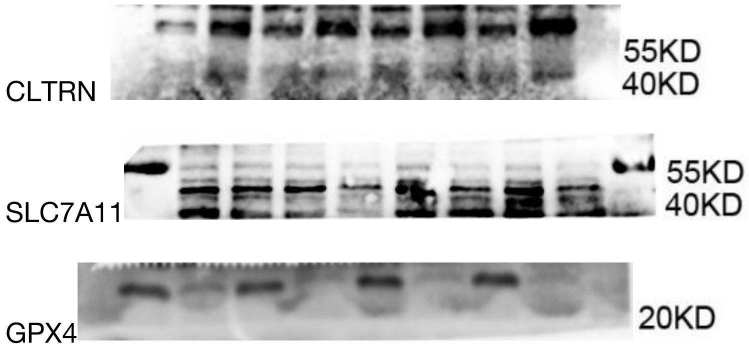

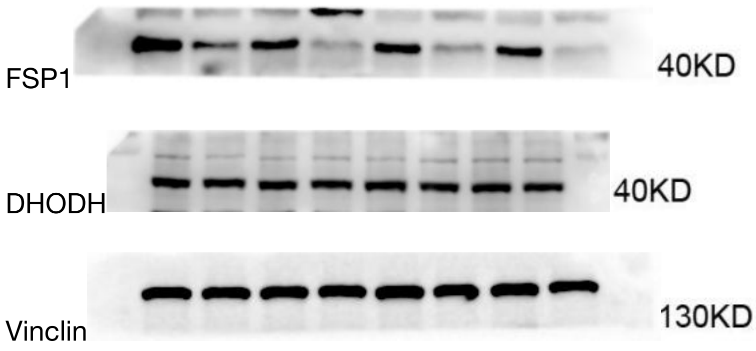

Figure6E Fadu

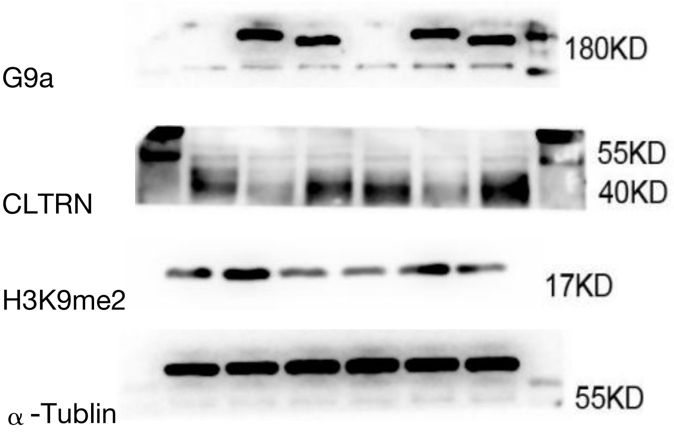

Figure6E HN8

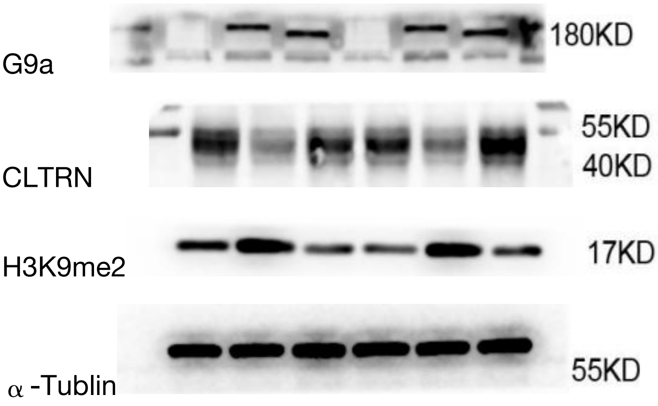

Figure7A

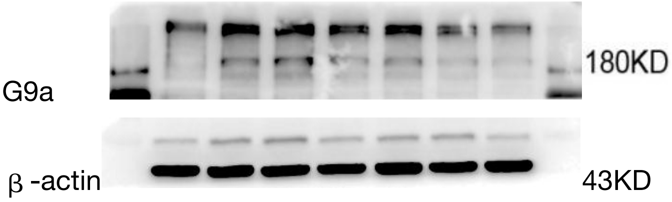

Figure7B Fadu

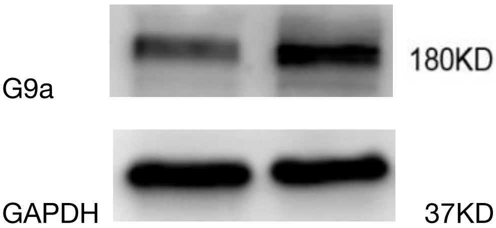

Figure7B HN8

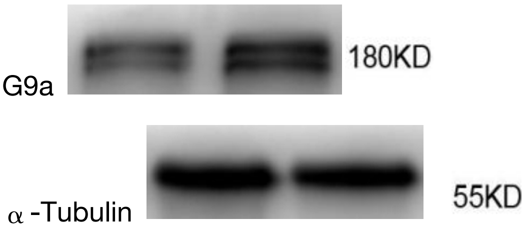

FigureS1A

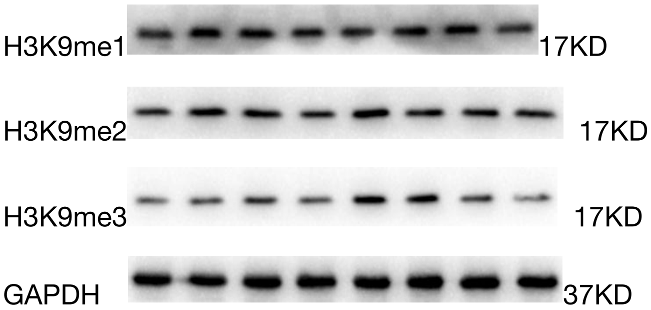

FigureS1B

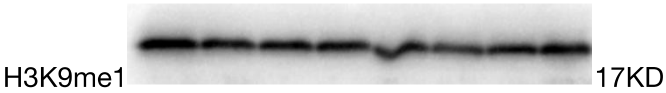

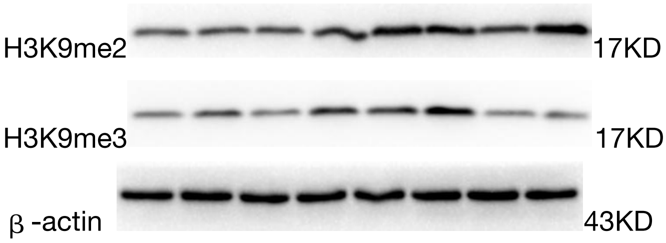

FigureS3A Fadu

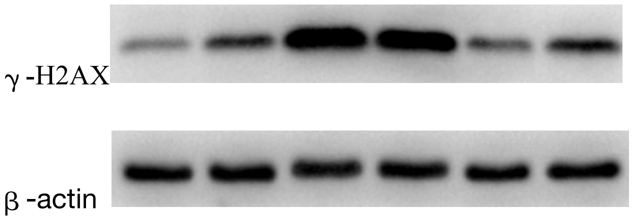

FigureS3A HN8

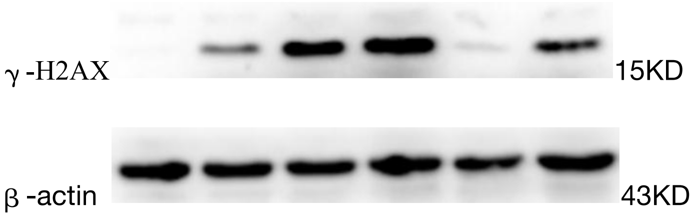

FigureS4C Fadu

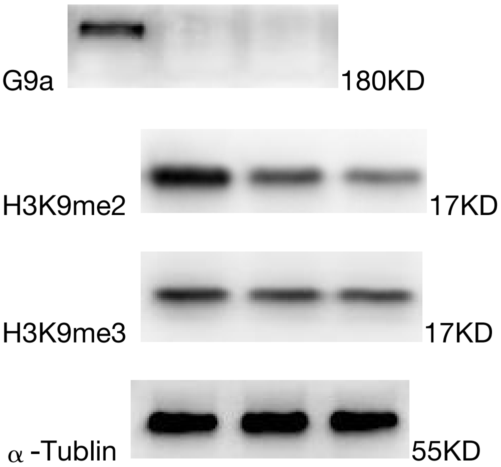

FigureS4C HN8

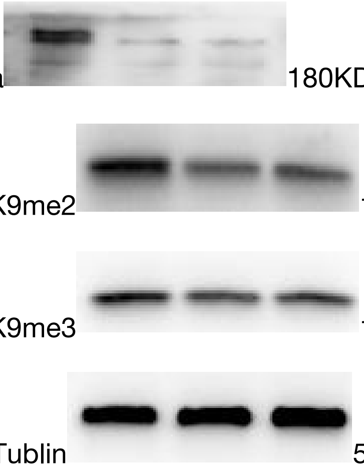

G9a 180KD

H3K9me2 17KD

H3K9me3 17KD

α -Tublin 55KD

FigureS6A

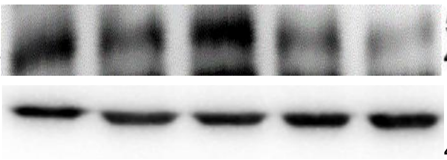

TMEM27 55KD 40KD

β -actin 43KD

FigureS6B Fadu

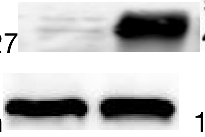

TMEM27 55KD 40KD

Vinculin 130KD

FigureS6B HN8

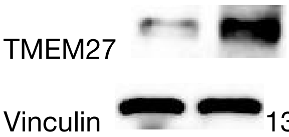

TMEM27 55KD 40KD

Vinculin 130KD
